# Caffeine and 4‐Hydroxybenzoic Acid Cocrystal Hydrate: Preparation, Characterization, and Evaluation

**DOI:** 10.1002/cphc.70545

**Published:** 2026-08-25

**Authors:** Shagun Gogna, Jonathan J. Du, Paul W. Groundwater, Mohamed Fares, Philip Chi Lip Kwok, Grace Tsz Yan Yau, Jan‐Willem C. Alffenaar, David E. Hibbs

**Affiliations:** ^1^ Sydney Pharmacy School Faculty of Medicine and Health The University of Sydney Camperdown New South Wales Australia; ^2^ Sydney Institute for Infectious Diseases The University of Sydney Sydney New South Wales Australia; ^3^ Westmead Hospital Sydney New South Wales Australia

**Keywords:** crystal growth, 4‐hydroxybenzoic acid, caffeine, dissolution, X‐ray diffraction

## Abstract

Caffeine is one of the most consumed stimulants with pharmaceutical benefits; however, its varied pH‐dependent solubility (120–‍500 µg/mL) and moderate stability hinder bioavailability. In this study, a 1:1:1 cocrystal hydrate of caffeine with 4‐hydroxybenzoic acid was crystallized and systematically reevaluated. SCXRD and PXRD revealed a triclinic system sustained by O─H···O and C─H···O hydrogen bonds. The Hirshfeld surface showed increased H···O/O···H interactions from 11.0% in caffeine to 15.7% in CAF·4HBA·H_2_O. Thermal analysis of CAF·4HBA·H_2_O showed a sharp melting endotherm at 166.7 °C with only a small mass loss (~2.46%) before melting, indicating high purity and good thermal stability. Dissolution studies showed the modulated caffeine release, with 2.8% released initially in pH 1.2, gradually increasing to 89.2% by 60 min and nearly complete release by 120 min. In pH 6.8, the CAF·4HBA·H_2_O achieved rapid and nearly complete release of caffeine within 60 min, compared to slightly slower release from pure caffeine. These results indicate that CAF·4HBA·H_2_O modulates dissolution kinetics rather than substantially increasing solubility. The present work emphasizes cocrystallization as a viable strategy to tailor dissolution behavior and stability, providing physicochemical insights relevant to oral formulation design.

## Graphical abstract

A 1:1:1 hydrate of caffeine (CAF) and 4‐hydroxybenzoic acid (4HBA) was crystallized to modulate drug performance. Structural analysis reveals enhanced intermolecular interactions, while dissolution studies show controlled release in gastric and rapid release in intestinal conditions. The study highlights cocrystallization as a strategy to tailor drug dissolution and stability.

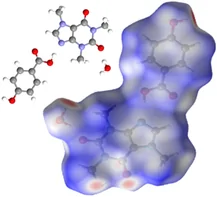

## 1 Introduction

The concept of cocrystallizing active pharmaceutical ingredients (APIs) with pharmaceutically acceptable coformers has become a preferred crystal engineering technique to control factors such as solubility, dissolution rate, mechanical behavior, and solid‐state stability without any change in the covalent structure of the drug molecule [1, 2]. Pharmaceutical cocrystals have become an attractive option in recent years as feasible alternatives to salts and polymorphs to enhance drug performance and to create new intellectual property possibilities [3, 4, 5]. Rational design of cocrystals is based on the understanding of intermolecular interactions and hydrogen‐bonding synthons of complementary functional groups of the API and the coformer [6, 7, 8]. This manipulation of intermolecular forces allows the formation of novel solid forms with improved physicochemical properties, and thus, cocrystals represent a promising field in the pharmaceutical development [9].

However, the result of cocrystallization is not always as expected and sometimes leads to the serendipitous discovery of alternative solid forms such as polymorphs [10, 11, 12, 13], solvates [13, 14, 15], and hydrates [13, 16, 17]. Although the contribution of coformers to the formation of new solid forms has not been clearly understood so far, recent literature has reported several such cases involving novel forms of APIs. Cocrystal hydrates are another type of solid form that is commonly found in cocrystallization experiments, but mainly because water is capable of engaging in many hydrogen‐bonding interactions in the crystal structure [18, 19].

**FIGURE 1:**
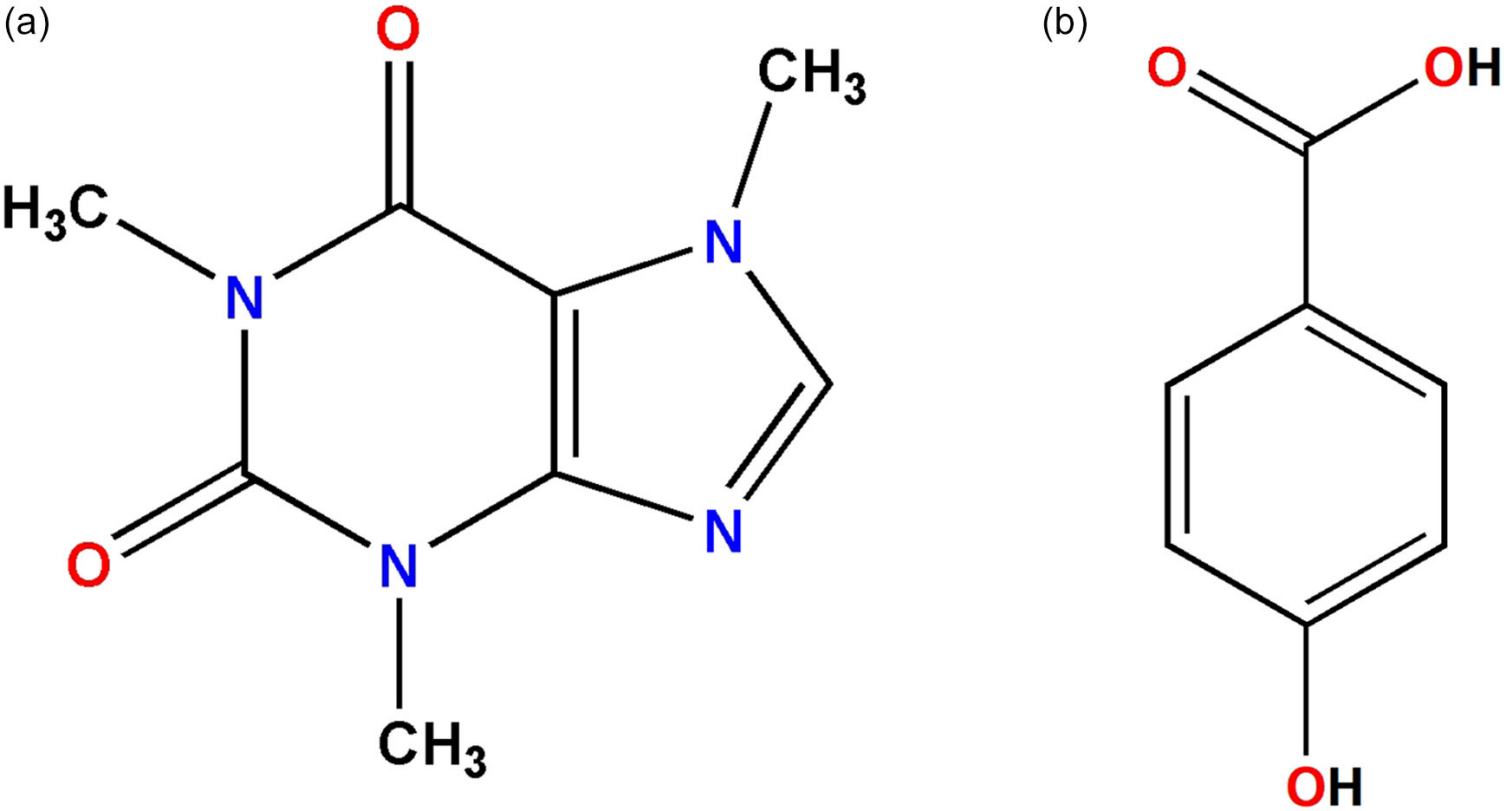
Chemical structures of (a) CAF and (b) 4HBA.

Caffeine (CAF, Figure 1a) is a commonly occurring central nervous system stimulant of the methylxanthine group [20]. It is found in several solid forms, two anhydrous polymorphs and a nonstoichiometric hydrate [21, 22]. Given these characteristics, CAF has several hydrogen bond acceptor sites, such as carbonyls and an imidazole nitrogen atom, which makes it an excellent candidate for cocrystal formation with appropriate hydrogen bond donors [23, 24]. The 4‐hydroxybenzoic acid (4HBA, Figure 1b) is particularly appealing due to its complementary functional groups such as carboxylic acid and phenolic hydroxyl groups, which can engage in predictable O─H···N and O─H···O hydrogen‐bonding interactions, respectively [25, 26].

Among all the CAF cocrystals, those with 4HBA have been comprehensively studied experimentally and computationally [27, 28]. Previous studies on the CAF·4HBA system have reported the formation of cocrystals with varying stoichiometries, highlighting the complex interplay between molecular recognition, crystallization conditions, and supramolecular assembly [26]. The carboxylic acid group of 4HBA typically forms a robust O─H···N hydrogen bond with the imidazole nitrogen of CAF, representing a reliable hetero‐synthon frequently observed in acid–amide or acid–heterocycle systems [27]. However, changes to the experimental conditions including solvent, concentration, crystallization method, etc. may change the stoichiometry and structure of the packing, sometimes resulting in a different solid form, such as a polymorph or hydrate [25, 26].

Surprisingly, cocrystallization with a 1:1:1 molar ratio of CAF and 4HBA has yielded the concomitant formation of 1:2 and 2:1 cocrystals [27, 28]. Computational studies by Price and coworkers found that a 1:1:1 cocrystal hydrate of CAF and 4HBA was predicted to be experimentally more stable than the 1:2 and 2:1 cocrystals [29]. Later, cocrystals between CAF and 4HBA with 1:2 and 2:1 stoichiometries were studied by pulsed gradient spin‐echo nuclear magnetic resonance, and they indicated that the ultimate cocrystal stoichiometry depends on the initial concentrations of the constituent solutions [28].

The experimental identification of a 1:1:1 cocrystal hydrate, supported by theoretical predictions of its thermodynamic stability, motivated a systematic investigation into the cocrystallization of CAF and 4HBA under varied experimental conditions [25, 29]. Although a 1:1:1 cocrystal hydrate phase was previously documented by Aitipamula *et al.* as a “hidden” structural intermediate, our work provides an extensive characterization of the structural and physicochemical properties governing this lattice [25].

Despite previous investigations, a comprehensive study focusing on the systematic preparation of CAF·4HBA·H_2_O, its structural characterization, and physicochemical analysis are of interest. The hydrogen‐bonding motifs and crystal packing characteristics, and with their effects on the bulk properties of the system including thermal characteristics and dissolution behavior, are areas essential for assessing the pharmaceutical relevance of this system.

In this work, we reevaluated the preparation of the caffeine‐4‐hydroxybenzoic acid cocrystal hydrate by solvent evaporation. We characterized the obtained solid form using single‐crystal and powder X‐ray diffraction (PXRD) as well as complementary thermal and spectroscopic methods. Furthermore, we evaluated its physicochemical properties in comparison with the parent components to assess potential improvements in performance. The study provides insight into the supramolecular interactions governing the CAF·4HBA·H_2_O system and highlights the role of crystal engineering in tailoring drug properties.

## 2 Materials and Methods

### 2.1 Materials

CAF (purity ≥ 98%), 4HBA (purity = 98%), and methanol were all purchased from Merck (Castle Hill, NSW) and used without further purification.

### 2.2 Experimental Methods

#### 2.2.1 Crystal Growth

A cocrystal hydrate of CAF and 4HBA was obtained by slow evaporation in a single‐solvent system. A 1:1 molar ratio of CAF and 4HBA was dissolved in methanol, as this ratio is commonly used in cocrystallization studies involving CAF and is known to produce stable cocrystals. Solutions were left to evaporate at room temperature, facilitating cocrystallization. This process resulted in the formation of a CAF·4HBA·H_2_O (1:1:1) cocrystal hydrate, as confirmed by subsequent characterization. Single crystal was selected using a polarized light microscope and mounted on thin glass fibers for X‐ray analysis.

#### 2.2.2 Single‐Crystal X‐Ray Diffraction Analysis

Single‐crystal X‐ray diffraction experiments were performed to determine the precise molecular and crystal structure of the CAF·4HBA·H_2_O, which is essential for understanding its intermolecular interactions and overall packing arrangement. These experiments were carried out at the Sydney Analytical X‐ray Diffraction Facility, University of Sydney, using a Rigaku Synergy Custom diffractometer with Cu *K*α radiation (*λ* = 1.54184 Å) at 150 K. Crystal of CAF·4HBA·H_2_O was mounted and cooled using an Oxford Cryosystems cooler. Data were acquired using 0.5° ω‐scans over a *θ* range of 3.26°–72.26°, corresponding to a high‐resolution limit of 0.81 Å. The reciprocal space was thoroughly covered by varying detector and φ‐scan positions to achieve 99% completeness. Multiscan absorption corrections were applied, with transmission factors ranging from 0.9511 to 0.9863. Exposure times were optimized for each scan range to ensure good signal‐to‐noise ratios. Absorption correction and data reduction were carried out using CrysAlisPro [30]. The structures were solved using SHELXT and refined by full‐matrix least‐squares on *F* ^2^ (SHELXL) [31]. All nonhydrogen atoms were refined anisotropically; hydrogen atoms were located from the difference map and were freely refined. Water hydrogen atoms were constrained to a fixed distance of 0.96 Å. Structural illustrations were generated using either Olex2 [32] or Mercury [33].

#### 2.2.3 PXRD Analysis

PXRD is commonly used to distinguish different crystal forms of a substance and is an effective technique for characterizing cocrystals [34]. PXRD measurements were performed to confirm the crystalline phase and purity of the synthesized CAF·4HBA·H_2_O. The samples were gently ground into fine powders and loaded onto a zero‐background silicon sample holder to minimize background noise. The measurements were performed on a Rigaku SmartLab diffractometer equipped with an X‐ray generator using Cu Kα radiation (*λ* = 1.54184 Å) generated at 40 kV and 40 mA. The sample was analyzed using reflection geometry with an ASC‐48 attachment and a standard goniometer. Data were collected in 1D scan mode over a 2*θ* range of 10° to 50°, with a scan speed of 10° per minute and a step width of 0.01°. The scan axis was set at *θ*/2*θ*. The incident slit was set to ½ degree, and a length‐limiting slit of 10 mm was applied. The detector used was an XSPA‐400 ER in horizontal setting mode, with an optics attribute, and no additional filters or slits were used. The diffraction patterns of the cocrystal hydrate were compared against those of the pure components to identify characteristic peak shifts or new reflections indicative of cocrystal hydrate formation [35]. Data analysis and phase identification were performed, confirming successful formation and phase purity of the CAF·4HBA·H_2_O.

#### 2.2.4 Thermal Test

The samples (CAF, 4HBA, and cocrystal hydrate), their thermal behavior, and melting point were evaluated using an Optimelt Automated Melting Point System. Around 4–6 mg of each material were examined at temperatures ranging from 25 to 400 °C in a melting point capillary tube. The heating rate was set to 1 °C/min, and each sample was analyzed in triplicate to ensure reproducibility. The reported melting points represent the average of the three runs, with onset and completion temperatures recorded for each [25].

Differential scanning calorimetry (DSC) measurements were performed using a Mettler Toledo DSC 1 model equipped with the STARe System (Mettler Toledo, Zürich, Switzerland). Thermogravimetric analysis (TGA) was carried out using a Mettler Toledo TGA/DSC 1 model with the STARe System (Mettler Toledo, Zürich, Switzerland). For both analyses, samples weighing approximately 3–10 mg were placed in aluminum sample pans and heated from 25 to 200 °C at a constant heating rate of 10 °C/min under a nitrogen atmosphere with a flow rate of 20 mL/min. The thermal data were analyzed using STARe software.

#### 2.2.5 Nuclear Magnetic Resonance Analysis (^1^H NMR)

The ^1^H NMR spectrum of the CAF·4HBA·H_2_O was recorded in DMSO‐*d*_6_ using a 300 MHz Bruker spectrometer (Bruker, Germany) at room temperature [36]. All spectra were processed using MestReNova software [37]. The spectrum was collected with 32 scans, a spectral width of 14.0 Hz, and a relaxation delay (d1) of 1.0 s [38].

#### 2.2.6 Hirshfeld Surface and 2D Fingerprint Plot Analysis

To study intermolecular interactions within the CAF·4HBA·H_2_O structure, Hirshfeld surface analysis was performed using *CrystalExplorer21* [39, 40]. This technique provided both visual and numerical insights based on crystal structure data obtained through single‐crystal X‐ray diffraction.

Hirshfeld surfaces were generated and mapped with respect to *d*_norm_, shape index, and curvedness. The surfaces were mapped using the *d*_norm_ parameter, which represents the normalized contact distance based on *d*_e_ and *d*_i_ [40]. Here, *d*_e_ is the distance from a point on the Hirshfeld surface to the nearest external atom, while *d*_i_ is the distance to the nearest internal atom. The *d*_norm_ surface was particularly useful for identifying intermolecular contacts—highlighting them with red (shorter than van der Waals radii), white (equal to van der Waals radii), and blue (longer) regions. The shape index was applied to detect π···π stacking patterns, while curvedness revealed the flatness or curvature of molecular regions, important for understanding stacking and surface topology [41, 42, 43].

Corresponding 2D fingerprint plots were created by plotting *d*_i_ (distance from the surface inward to the nearest nucleus) against *d*_e_ (distance outward to the nearest atom). These plots allowed quantification of interaction types, including H···H, O···H/H···O, N···H/H···N, and C···H/H···C, with their relative surface contributions calculated. This helped in evaluating how cocrystallization modified intermolecular contacts compared to the pure drug components. All surfaces were visualized using the default high‐resolution settings in *CrystalExplorer21* (surface resolution, 0.5 a.u.; *d*_norm_ color scale, −0.3 to 1.5 a.u.) [40].

#### 2.2.7 Lattice Energy Estimation

To assess the thermodynamic stability of the cocrystal hydrate, lattice energies were calculated using *CrystalExplorer21* [40, 44] which estimates intermolecular interaction energies based on the three‐dimensional arrangement of molecules within the crystal and CP2K which provides a fully quantum mechanical evaluation of lattice energies, incorporating contributions from exchange–correlation effects and basis set expansions.

Periodic density functional theory calculations were performed in CP2K using structures derived from CIF files, with input files generated *via* Multiwfn. A K‐point convergence analysis was conducted to ensure reliable Brillouin zone sampling, and the optimized K‐point grid was used throughout. Geometry optimizations were carried out under periodic boundary conditions employing a DZVP basis set in conjunction with Grimme’s D3‐BJ dispersion correction. Subsequently, single‐point energies were calculated using a higher level TZVP basis set to improve energetic accuracy. Gas‐phase calculations of the individual molecular components (API and coformer), extracted from the asymmetric unit, were performed without periodic boundary conditions at the same level of theory. The lattice energy was determined as the difference between the periodic crystal energy and the sum of the gas‐phase molecular energies, normalized per formula unit and converted to kJ/mol.

Complementary calculations were performed in *CrystalExplorer21* to analyze intermolecular interactions and energy frameworks. These calculations used the experimental CIF files directly, maintaining the original packing arrangement without additional geometry optimization, ensuring that the energy evaluation accurately reflects the experimentally observed crystal environment [45].

Each interaction energy (*E*_tot_) included contributions from four components: electrostatic (*E*_ele_), polarization (*E*_pol_), dispersion (*E*_disp_), and exchange‐repulsion (*E*_rep_). These were calculated using molecular wavefunctions based on the B3LYP/6‐31G(d,p) method, and the interaction energy was evaluated within a 3.8 Å radius surrounding each molecule. Three‐dimensional energy frameworks were also constructed. In these, interaction strengths were visualized as cylinders between molecular centroids, with the cylinder thickness corresponding to the magnitude of interaction [45]. These frameworks allowed clear comparison of packing efficiency and cohesive forces across different cocrystals and their original components. All procedures were carried out using standard settings recommended for pharmaceutical cocrystal studies in *CrystalExplorer21* [46].

More negative lattice energy values obtained indicate stronger overall cohesive forces within the crystal lattice and hence greater stability of the cocrystal. The comparison between *CrystalExplore*r*21* and CP2K results enables validation of intermolecular interaction trends and provides deeper insight into the energetic landscape governing cocrystal formation.

#### 2.2.8 High‐Performance Liquid Chromatography (HPLC)

Quantification of CAF alone was performed using an Agilent 1200 HPLC system equipped with an Agilent G1311A pump and G1315D diode‐array detector (DAD). Chromatographic separation was achieved on a C18 reverse phase column (250 mm × 4.6 mm, 5 µm particle size) maintained at 30 °C, with a mobile phase of sodium acetate buffer and acetonitrile in a 90:10 ratio. The flow rate was set to 1 mL/min, and the detection wavelength was 240 nm. The retention time for CAF was 7.8 min [47]. This analysis was conducted to determine the concentration of free CAF remaining in samples during dissolution or stability studies, allowing assessment of the extent of cocrystal hydrate formation and the proportion of unconverted CAF. By quantifying free CAF, the efficiency of cocrystallization and the release profile of CAF from the CAF·4HBA·H_2_O could be evaluated.

#### 2.2.9 Solubility Studies

CAF, its physical mixture with 4HBA, and the CAF·4HBA·H_2_O were evaluated for solubility using a modified shake‐flask method [48]. Excess amounts of each sample (equivalent to 50 mg of CAF) were added to 10‐mL aliquots of 0.01 N HCl buffer (pH 1.2 ± 0.2) and phosphate buffer (pH 6.8 ± 0.2). Samples were incubated at 37 ± 0.5 °C with continuous shaking at 100 rpm for 2 h. The supernatant was filtered through 25‐mm syringe filters with a nylon membrane of 0.22 µm pore size and analyzed *via* HPLC [49].

#### 2.2.10 Drug Content Analysis

A stock solution of CAF (1 mg/mL) was prepared by dissolving 100 mg of pure CAF in 100 mL of phosphate buffer (pH 6.8) using a volumetric flask. This standard stock solution was further diluted with phosphate buffer to obtain a working standard solution of 100 µg/mL. Subsequent serial dilutions of this working solution were made to prepare standard solutions in the concentration range of 2–16 µg/mL. The absorbance of each solution was measured at the *λ*_max_ of 240 nm using a UV spectrophotometer, and a calibration curve was constructed. Statistical parameters including the slope, intercept, and correlation coefficient (*R* ^2^) were determined to assess linearity.

For drug content analysis, 10 mg of cocrystal hydrate sample was accurately weighed and dissolved in phosphate buffer (pH 6.8) to yield a final concentration of 10 µg/mL. The absorbance of each sample was measured at 240 nm, and the CAF content was quantified using the calibration curve [50].

#### 2.2.11 *In Vitro* Simulated Gastrointestinal Digestion

To explore the kinetics of drug release in gastrointestinal fluid, the release profile of CAF from the CAF·4HBA·H_2_O was studied under sequential simulated gastric and intestinal conditions [51]. Approximately 30 mg of the CAF·4HBA·H_2_O (equivalent to ~17.5 mg of CAF) was added to 100 mL of simulated gastric fluid (SGF; 0.01 N HCl, pH 1.2) and stirred at 37 °C with constant agitation (100 rpm) for 2 h. After this gastric phase, the pH was adjusted to 6.8, and the medium was mixed with simulated intestinal fluid (SIF; ‍phosphate buffer, pH 6.8) in a 1:1 (v/v) ratio, followed by continued stirring at 37 °C for an additional 2 h. During the entire digestion process, 1‐mL aliquots were withdrawn and immediately replaced with 1 mL of fresh, prewarmed medium to maintain sink conditions and constant volume. The concentration of CAF in the collected samples was determined *via* HPLC (*n* = 3).

## 3 Results and Discussion

### 3.1 PXRD Analysis

**FIGURE 2:**
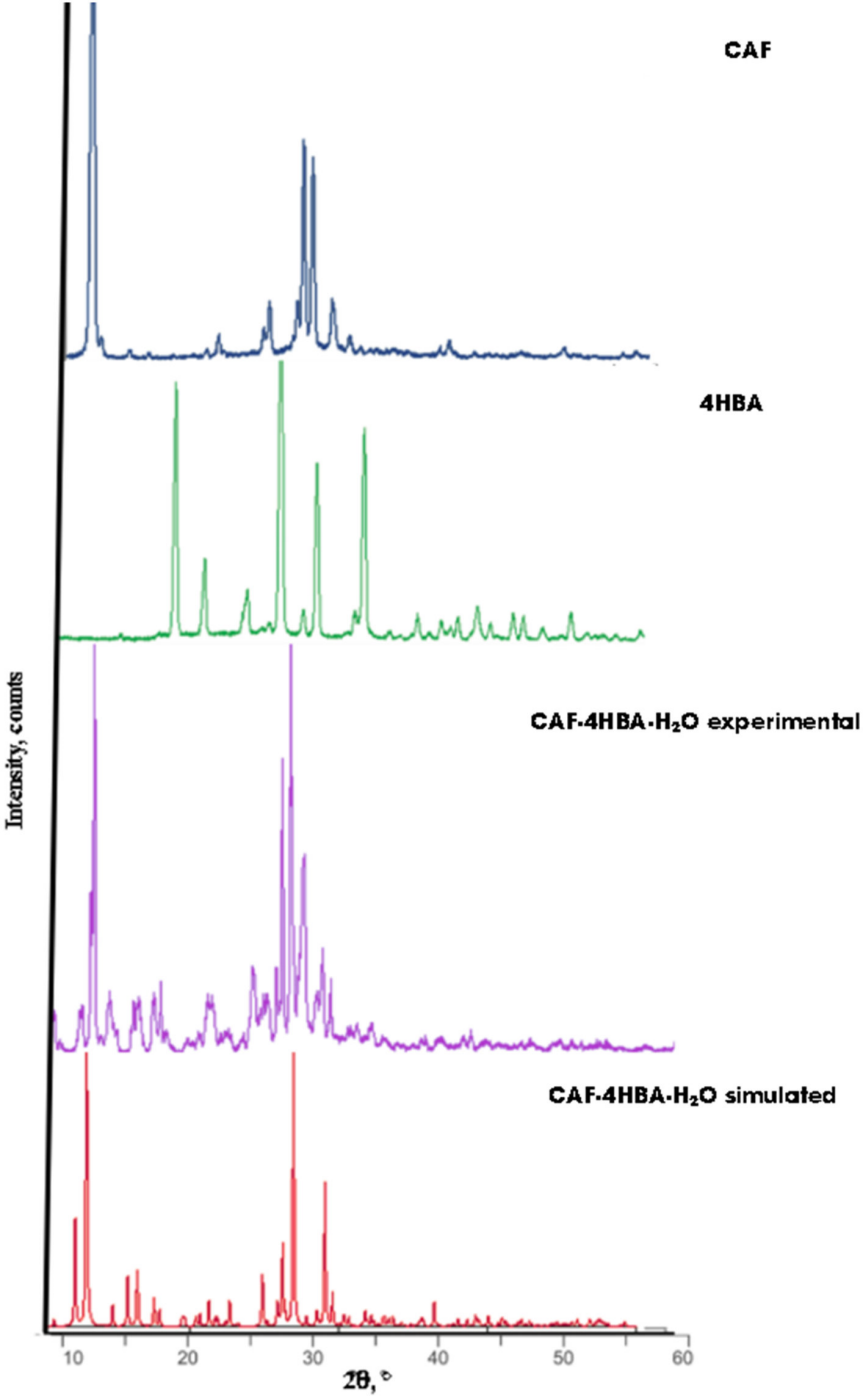
Comparative PXRD pattern vs. simulated diffraction pattern of CAF·4HBA·H_2_O.

The phase purity and crystalline nature of the CAF·4HBA·H_2_O were evaluated using PXRD. The PXRD pattern of the CAF·4HBA·H_2_O shows distinct diffraction peaks between 10° and 50° 2*θ* (Figure 2).

The individual components exhibit well‐defined Bragg reflections: Pure CAF displays characteristic peaks at 12.3°, 17.9°, 22.1°, 24.7°, and 27.5° (2*θ*), while 4‐hydroxybenzoic acid (4HBA) shows distinct reflections at 11.4°, 15.2°, 18.6°, 21.9°, and 26.3°. In contrast, the PXRD profile of the CAF·4HBA·H_2_O reveals the emergence of characteristic reflections at approximately 12.28°, 13.16°, 27.02°, and 29.15°. The complete disappearance of the characteristic parent reflections and the appearance of these new diffraction signals confirm the formation of a new crystalline phase, distinct from a physical mixture of the components.

According to the research by Aitipamula *et al.*, the CAF·4HBA·H_2_O system is prone to forming “hidden hydrates” [25]. Their work demonstrates that the 1:1:1 cocrystal hydrate phase acts as a critical structural intermediate; the PXRD pattern for this cocrystal hydrate is specifically defined by the water‐mediated lattice arrangement, which will be analyzed in the geometry section [25]. The experimental pattern obtained in this study matches the simulated diffraction pattern derived from the single‐crystal data, confirming the successful preparation of the previously reported phase‐pure 1:1:1 cocrystal hydrate [25].

The unique reflections observed are consistent with the long‐range order of the cocrystal hydrate lattice, where the water molecule facilitates the specific packing arrangement [25]. As noted by Aitipamula, any shift in these peaks upon heating would indicate the dehydration of the 1:1:1 cocrystal hydrate and a subsequent structural rearrangement into the anhydrous 1:2 or 2:1 cocrystal phases. The alignment between experimental PXRD and the single‐crystal simulation validates the successful preparation of the specific cocrystal hydrate architecture identified in the literature [25].

### 3.2 Thermal Test

The melting temperatures of 4HBA, CAF, and their cocrystal hydrate exhibit significant differences, highlighting the formation of a new crystalline phase. While pure 4HBA melts at 215.4–‍215.7 and CAF at 210.9–211.2 °C, the cocrystal hydrate shows a much lower melting point of 162.0–162.3 °C. This marked decrease in melting temperature for the cocrystal hydrate compared to its individual components suggests a distinct crystalline structure and confirms the presence of a new phase. The sharp endothermic peak observed for the cocrystal hydrate further emphasizes its unique thermal behavior and indicates high purity. In contrast to the higher melting points of the parent compounds, the lower melting temperature of the cocrystal hydrate reflects the altered molecular interactions between CAF and 4HBA.

**FIGURE 3:**
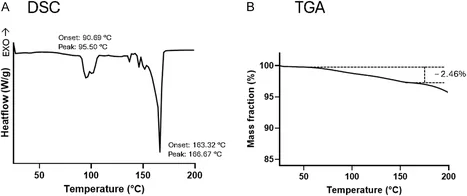
The thermal analysis patterns of CAF·4HBA·H_2_O.

DSC analysis investigated the thermal properties of the cocrystal hydrate. From the DSC profiles (Figure 3a), the cocrystal hydrate exhibited two endothermic events. The first broad endothermic peak appeared at 95.50 °C (onset: 90.69 °C), which may be attributed to the loss of weakly bound or lattice water molecules. A second sharp endothermic peak was observed at 166.67 °C (onset: 163.32 °C), corresponding to the melting of the cocrystal hydrate. The melting behavior differed from that of the parent components, confirming the formation of a new crystalline phase.

TGA was applied to investigate the decomposition process of the cocrystal hydrate. Based on the results of TG analysis (Figure ‍3b), the CAF·4HBA·H_2_O exhibited an initial mass loss of approximately 2.46% below 190 °C, which may be attributed to partial loss of lattice or adsorbed water. No significant decomposition was observed before the melting point, indicating that the cocrystal hydrate remained thermally stable up to approximately 160 °C. The gradual mass loss at higher temperatures corresponds to the thermal decomposition of the cocrystal hydrate components.

### 3.3 Proton Nuclear Magnetic Resonance (^1^H ‍NMR)

The ^1^H NMR spectra of CAF, 4HBA, and CAF·4HBA·H_2_O show distinct yet related features in solution. The spectrum of CAF exhibits characteristic resonances including a singlet for the heterocyclic proton at *δ* 7.96 (1H) and three *N*‐methyl signals appearing in the *δ* 3.17–3.84 region, consistent with its fully substituted xanthine structure. In contrast, 4HBA displays two doublets at *δ* 6.82 (2H) and *δ* 7.79 (2H), along with downfield signals attributable to the phenolic OH (*δ* 10.24 ppm) and the carboxylic acid OH (*δ* 12.35 ppm), in agreement with literature data for *para*‐substituted benzoic acids.

The ^1^H NMR spectrum of the CAF·4HBA·H_2_O displays resonances attributable to both CAF and 4HBA within a single spectrum, confirming the presence of both components in the isolated solid. The relative integrations of the CAF methyl and aromatic signals compared with those of 4HBA are consistent, indicating the formation of a defined multicomponent system rather than a random physical mixture. In addition, small chemical shift changes and broadening of aromatic and exchangeable OH resonances relative to the pure compounds suggest alterations in the local proton environments, consistent with intermolecular interactions such as hydrogen bonding. It should be noted that the ^1^H NMR spectra were recorded in solution; therefore, the solid‐state hydrogen‐bonding network present in the cocrystal hydrate may be partially or fully disrupted by solvent effects. As a result, the NMR data provide supportive evidence for cocrystal hydrate formation.

### 3.4 Geometry

**FIGURE 4:**
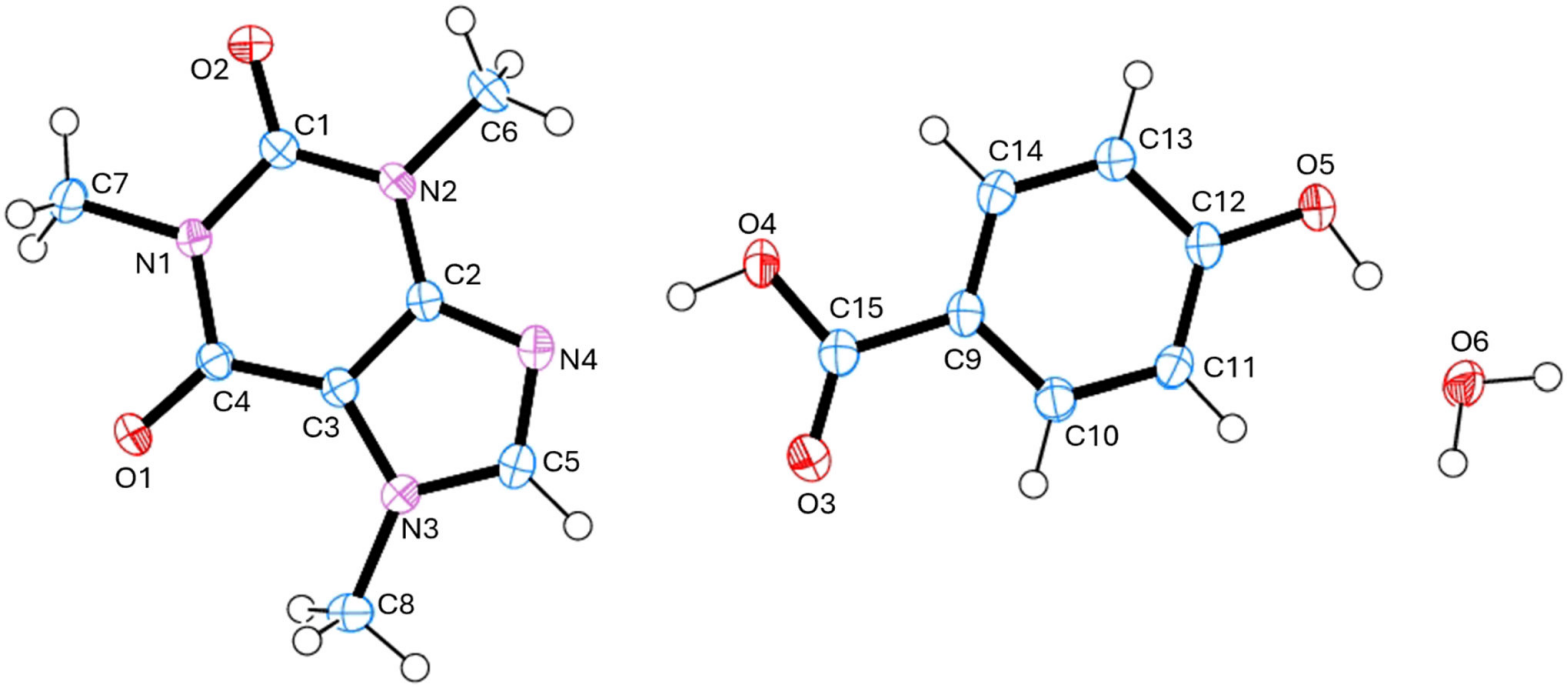
ORTEP diagrams of CAF·4HBA·H_2_O.

The crystal structure of the CAF·4HBA·H_2_O consists of CAF, 4‐hydroxybenzoic acid (4HBA), and one lattice water molecule in a 1:1:1 stoichiometric ratio which crystallizes in the triclinic space group. The structure is stabilized by a three‐dimensional hydrogen‐bonding network in which the water molecule plays a central bridging role between CAF and 4HBA molecules. The molecular geometries of CAF and 4HBA are consistent with previously reported crystal structures, with only minor variations in bond lengths and angles arising from intermolecular interactions within the crystal lattice [25]. The molecular structure and atomic labeling of the asymmetric unit are shown in Figure 4. By merging the geometric and hydrogen‐bonding descriptions into a unified analysis, it becomes evident that this stoichiometry is distinct from the previously reported 2:1 and 1:2 anhydrous cocrystals, which are typically favored under concentration‐dependent conditions in solution‐mediated phase transformations [27, 28].

The primary structure‐directing interaction is the classical carboxylic acid–imidazole hetero‐synthon formed between the carboxylic acid group of 4HBA and the imidazole nitrogen atom of CAF through an O4─H4···N4 hydrogen bond (d(D···A) = 2.645(2)Å, ∠D─H···A = 176(2)°). This interaction forms the principal supramolecular motif within the lattice and provides a rigid structural backbone for the assembly. The intrinsic geometry of lattice water molecule (O6) with an H6D–O6–H6E angle of 106.8(16)° and bond lengths 0.9598(11) Å for both (O6···H6D) and (O6···H6E) is characteristic of lattice‐bound water; its specific orientation within this lattice allows it to function as a double hydrogen‐bond donor and single hydrogen‐bond acceptor. It bridges neighboring molecules through O6─ H6D···O1 (at 2.678 Å) and O6─H6E···O2 (at 2.695 Å) hydrogen bonds involving the CAF carbonyl groups, while simultaneously accepting a hydrogen bond from the phenolic hydroxyl group of 4HBA through the O5─H5A···O6 (at 2.576 Å) interaction. These interactions generate a continuous water‐mediated hydrogen‐bonding network that stabilizes the cocrystal hydrate structure.

**FIGURE 5:**
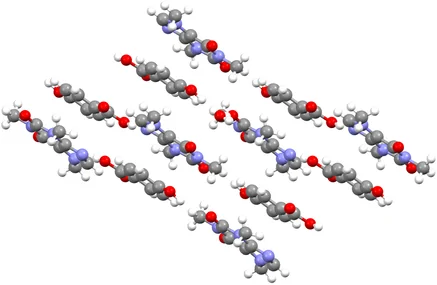
Crystal packing of CAF·4HBA·H_2_O viewed along the *b* axis. Molecules are connected *via* C─H···O and O─H···O hydrogen bonds, forming a three‐dimensional network. Visualization and analysis were performed using Mercury.

The observed C12–O5–H5A angle of 108.8(12)° further reflects the directed orientation of the phenolic hydroxyl group toward the lattice water molecule rather than a direct phenol–CAF interaction, highlighting the structural role of water in mediating the hydrogen‐bond network. This water‐mediated bridging arrangement differs from the direct phenol–CAF contacts observed in the anhydrous forms and effectively satisfies the hydrogen‐bonding requirements of both the phenolic donor and CAF carbonyl acceptors. Consequently, the lattice water molecule acts as a supramolecular “glue” that stabilizes the cocrystal hydrate framework and supports the otherwise less favored 1:1 stoichiometry proposed to be difficult to sustain in the absence of water [29]. Unlike the anhydrous 2:1 form, where 4HBA bridges multiple CAF sites directly, or the 1:2 form involving extended phenolic chain interactions, the present cocrystal hydrate utilizes the O6 water node to balance donor–acceptor interactions within the lattice [27, 29]. The resulting three‐dimensional supramolecular arrangement is illustrated in the crystal packing diagram (Figure 5).

The experimentally determined hydrogen‐bond geometries are consistent with the cocrystal hydrate structure previously reported by S. Aitipamula [25]. Minor variations in bond distances and angles are within expected crystallographic limits and likely reflect differences in data collection and refinement conditions rather than substantial structural differences. The hydrogen‐bonding parameters for the major intermolecular interactions are summarized in Table 1. In addition to the classical hydrogen bonds, weaker intermolecular contacts including C─H···O interactions contribute to the overall crystal packing. Together, these interactions form a three‐dimensional supramolecular network that stabilizes the crystal lattice of the CAF·4HBA·H_2_O.

**TABLE 1:** Comparison of hydrogen‐bond geometries (Å and °) for the 1:1:1 cocrystal hydrate to 2:1 and 1:2 CAF‐4HBA cocrystal [27] (with estimated standard deviations except for fixed and riding hydrogen atoms).

Interaction (D─H···A)	Phase	d(D─H), Å	d(H···A), Å	d(D···A), Å	∠D─H···A, °
O4─H4···N4	CAF·4HBA·H_2_O (1:1:1)	1.01(2)	1.72(2)	2.645(2)	176(2)
O6─H6D···O1	CAF·4HBA·H_2_O (1:1:1)	0.94(3)	1.75(3)	2.678(3)	165(3)
O6─H6E···O2	CAF·4HBA·H_2_O (1:1:1)	0.89(3)	1.81(3)	2.695(3)	168(3)
O5─H5A···O6	CAF·4HBA·H_2_O (1:1:1)	0.95(2)	1.63(2)	2.576(2)	177(2)
O4─H1X···N2 [27]	CAF‐4HBA (1:2)	0.90(4)	1.79(4)	2.689(3)	175(4)
O5─H3X···N8 [27]	CAF‐4HBA (2:1)	0.90(2)	1.79(2)	2.681(2)	168(2)

### 3.5 Hirshfeld Surfaces and 2D Fingerprint Plots

Hirshfeld surface analysis provides a detailed visualization of intermolecular interactions by highlighting both short and long contacts, with varying colors and intensities. The *d*_norm_ maps of the complex (Table 2) depict these interactions, where red and blue regions indicate contacts that are shorter or longer than the van der Waals radii, respectively, while the white surface represents distances equal to the sum of these radii. This method examined the structures of the cocrystals, with Hirshfeld surfaces and molecular fingerprints generated using *CrystalExplorer21* [40] to explore the interactions within each system. The analysis, performed at high resolution (isovalue 0.5), provided valuable insights into the molecular surface properties of each cocrystal, revealing essential information about their molecular characteristics and intermolecular interactions. The results emphasize the nature of contact regions and packing arrangements, contributing to a deeper understanding of their structural and interactional features.

**TABLE 2:** Comparative Hirshfeld surface and topological analysis of CAF and CAF·4HBA·H_2_O.

Plots	CAF	4HBA	CAF·4HBA·H_2_O
Hirshfeld surface	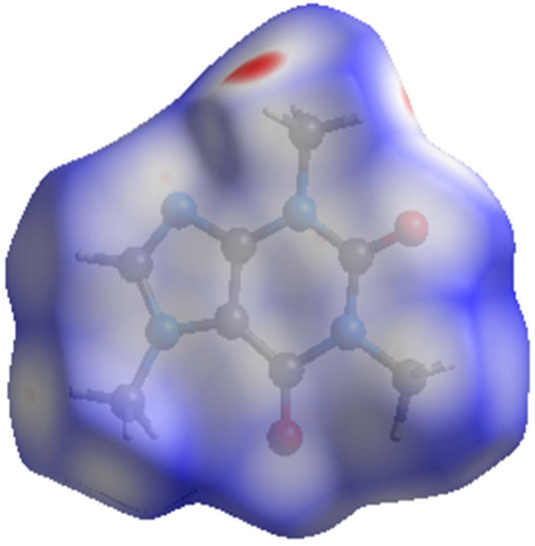	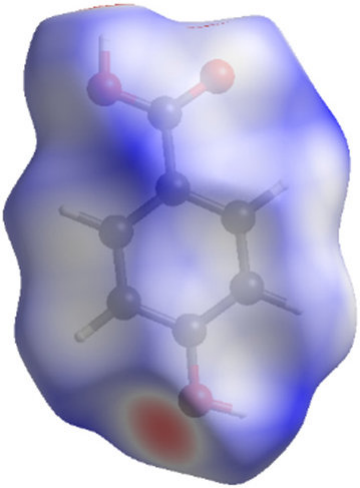	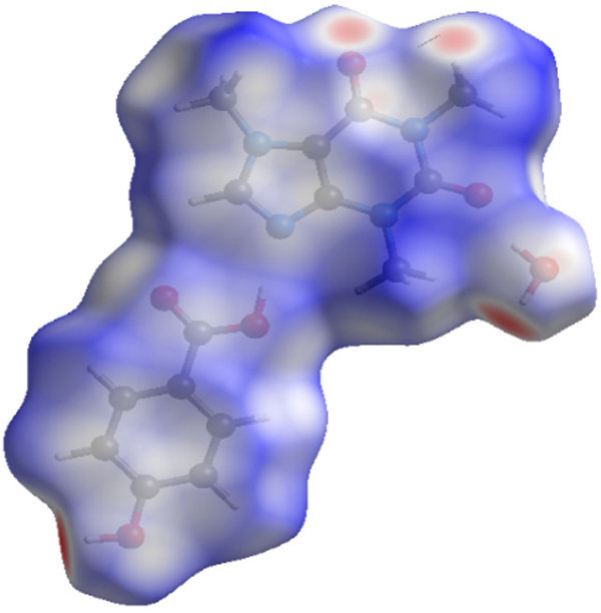
Shape index plot	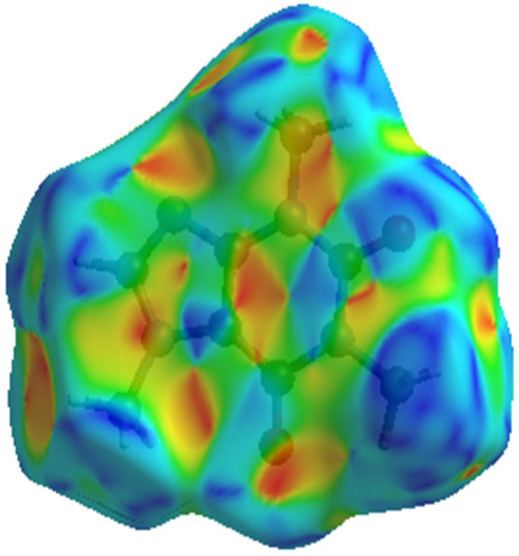	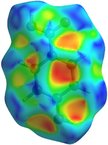	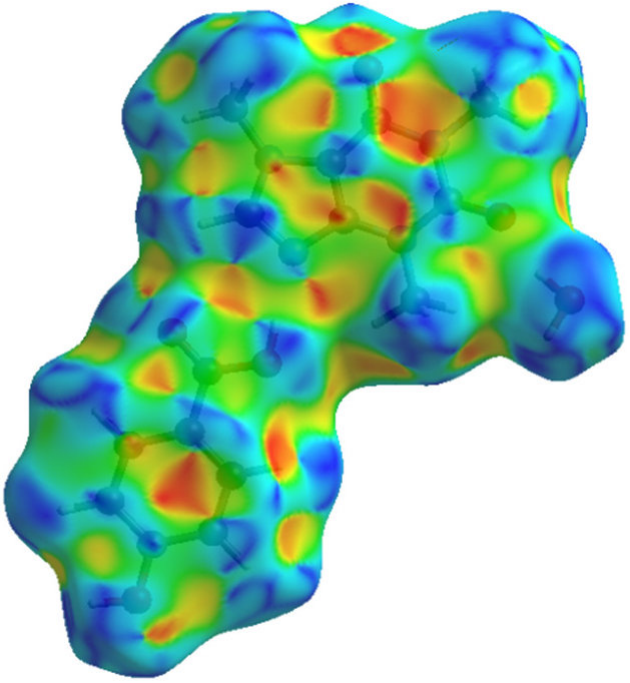
Curvedness mapping	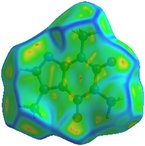	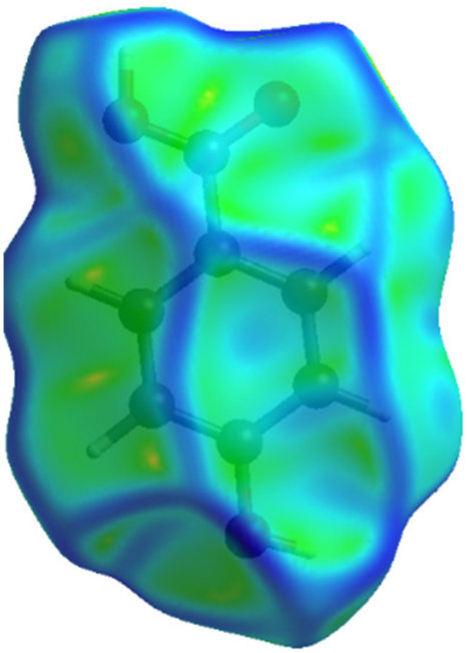	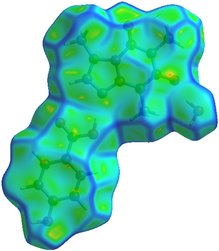
Fragment Patch	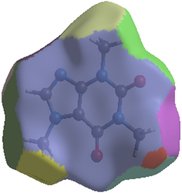	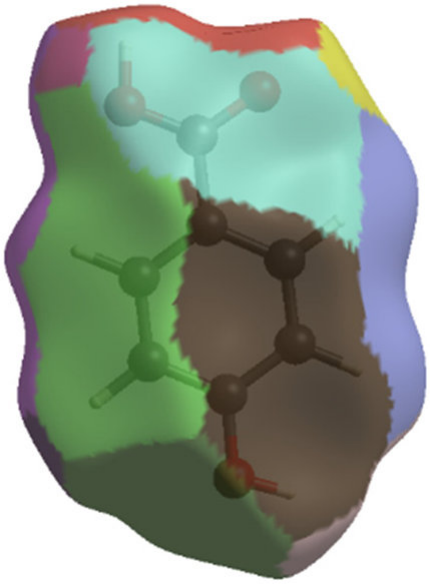	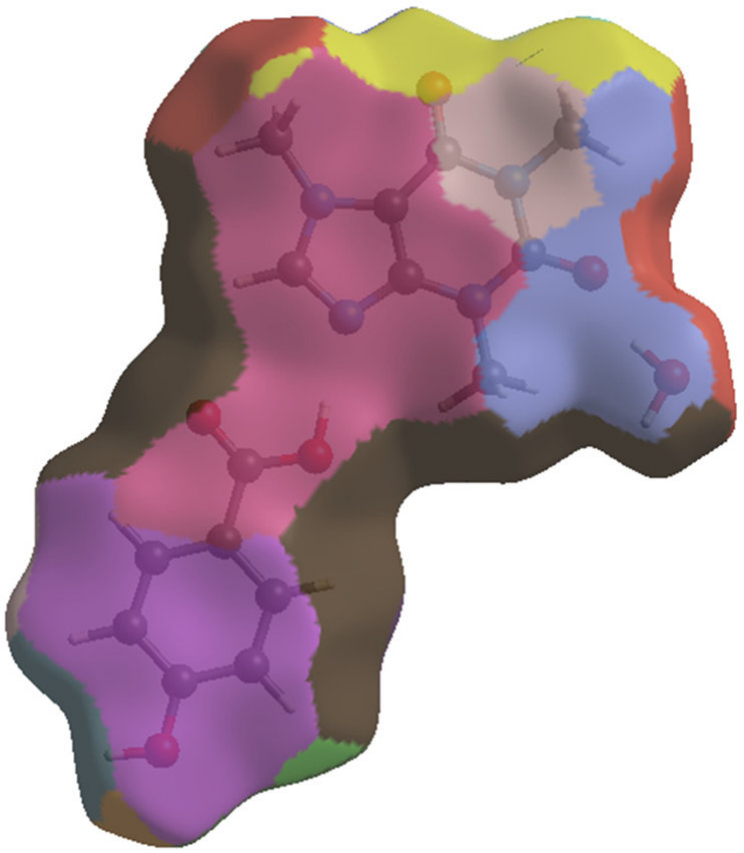

The comparison between CAF and CAF·4HBA·H_2_O reveals significant differences in molecular surface characteristics and interaction profiles. CAF exhibits a smaller molecular volume (220.99 Å^3^) and surface area (221.61 Å^2^) compared to CAF·4HBA·H_2_O (368.00 Å^3^ and 355.84 Å^2^, respectively), reflecting the increased molecular complexity upon cocrystal hydrate formation. CAF also demonstrates higher globularity (0.798) and lower asphericity (0.121), indicating a more compact and spherical structure than CAF·4HBA·H_2_O (globularity, 0.698; asphericity, 0.366), which is more elongated and irregular. The *d*_i_ and *d*_norm_ ranges in CAF·4HBA·H_2_O are broader and more negative (*d*_i_, 0.639–2.342 Å; *d*_norm_, −0.757 to 1.241) than those of CAF (*d*_i_, 0.924–2.292 Å; *d*_norm_, −0.263 to 1.259), suggesting stronger and more diverse intermolecular interactions in the cocrystal hydrate. Additionally, both the shape index and curvedness values in CAF·4HBA·H_2_O span a wider range, indicating a more complex and varied molecular surface topology. These distinctions collectively highlight the structural and interactional enhancements introduced through cocrystal hydrate formation.

In the shape index surfaces, complementary red and blue triangular regions are observed, indicating close aromatic–aromatic contacts within the crystal packing. These features suggest the presence of π‐associated interactions between the aromatic systems of 4‐hydroxybenzoic acid and the fused heteroaromatic framework of CAF. However, interactions are not assigned here to specific subunits of CAF ring system due to the fused nature of its purine framework [42].

Relatively large green planes appear where the benzene and pyridine ring ligands are situated. The curvedness surface further highlights relatively flat regions associated with planar stacking arrangements, consistent with aromatic stacking interactions contributing to crystal stability (Table 2) [42].

The Fragment Patch surface (Table 2) shows complementary contact regions between neighboring molecules, indicating efficient surface matching during crystal packing. These results collectively demonstrate the presence of stabilizing noncovalent interactions, including hydrogen bonding and aromatic contacts, which govern the overall packing architecture of the cocrystal hydrate.

### 3.6 Fingerprint Plot Analysis

The Hirshfeld surface analysis of CAF and CAF·4HBA·H_2_O reveals distinct shifts in intermolecular interactions upon cocrystal hydrate formation, underscoring the structural influence of the 4HBA coformer. In both structures, H···H contacts remain the most dominant interaction, contributing 66.9% of the total surface, indicating that van der Waals forces are a key feature in both packing arrangements. However, CAF·4HBA·H_2_O shows a notable increase in H···O/O···H interactions (15.7%) compared to CAF (11.0%), reflecting enhanced hydrogen bonding *via* the ─OH and ─COOH groups of 4HBA. Additionally, H···C/C···H contacts increase from 10.2% in CAF to 13.5% in CAF·4HBA·H_2_O, suggesting new C─H···π or related interactions introduced by the coformer. Conversely, H···N/N···H interactions decrease significantly in CAF·4HBA·H_2_O (3.8%) compared to CAF (11.9%), indicating a reduced role of nitrogen‐based hydrogen bonding, likely due to the redistribution of interaction sites. Minor contacts such as N···C/C···N (3.5%) and O···C/C···O (0.1%) in CAF shift to a small C···O/O···C contribution (0.9%) in the cocrystal hydrate. Importantly, C···C contacts increase from 4.5% in CAF to 6.3% in CAF·4HBA·H_2_O. This increase suggests enhanced aromatic–aromatic proximity within the crystal packing. These changes, visualized as deeper red regions on the d_norm_‐mapped Hirshfeld surfaces, confirm that cocrystallization alters the balance and type of interactions, enhancing O─H···O and N─H···O hydrogen bonding in CAF·4HBA·H_2_O. Full 2D fingerprint plots and individual interaction profiles are summarized in Table 3.

**TABLE 3:** 2D fingerprint plots of (a) CAF, (b) 4HBA, and (c) CAF·4HBA·H_2_O.

	CAF	4HBA	CAF·4HBA·H_2_O
Overall	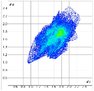	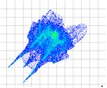	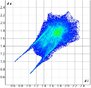
H···H	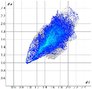	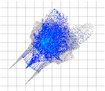	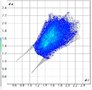
O···H/H···O	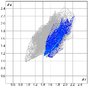	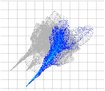	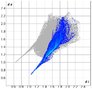
C···H/H···C	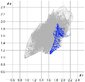	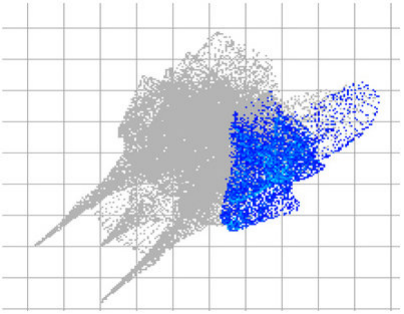	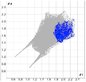
N···H/H···N	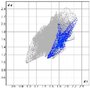	—	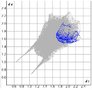
C···C	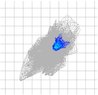	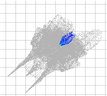	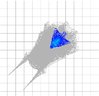

### 3.7 Lattice Energy

To assess the thermodynamic stability of the CAF‐4HBA 1:1:1 cocrystal hydrate, lattice energies were evaluated using two complementary computational approaches: periodic density functional theory *via* CP2K and pairwise intermolecular interaction energy summation *via* *CrystalExplorer21* [40]. This dual‐methodology approach allows for a distinction between local supramolecular strengths and the global stability of the extended crystal lattice [29, 52].

The reference molecule’s total intermolecular energy, *E*_tot_ (kJ/mol), is the sum of its four primary energy components: electrostatic (*E*_ele_), polarization (*E*_pol_), dispersion (*E*_disp_), and exchange‐repulsion (*E*_rep_). Scale factors were applied to each component (*E*_ele_, *E*_pol_, *E*_disp_, and *E*_rep_) based on reported values [46, 53] to improve the accuracy of the intermolecular energy calculations. The energy calculations were performed using the B3LYP/6‐31G(d,p) [54] model in *CrystalExplorer21* [40]. The results show that the dispersion (*E*_disp_), polarization (*E*_pol_), and exchange‐repulsion (*E*_rep_) energy values remained consistent across both models, reflecting the stability of the reference molecule. In contrast, the electrostatic energy (*E*_ele_) values varied, which had a noticeable effect on the total energy. Previous research has indicated that hydrogen bond interactions, such as N─H···O and C─H···O, are key contributors to these energies within the complex’s crystal structure. The stability of the dispersion energy across the models suggests that the molecule remains stable, as specific hydrogen–hydrogen (H···H) interactions identified through Hirshfeld surface analysis significantly enhance its stability [55].

The periodic CP2K calculation, which treats the crystal as an infinite lattice under periodic boundary conditions, yielded a lattice energy of kJ/mol. In contrast, the *CrystalExplorer* method, which sums the interaction energies of molecular pairs within the first coordination shell, resulted in a total interaction energy of kJ/mol. The significantly more negative value obtained from the CP2K model is attributed to its ability to account for the collective polarization and long‐range cohesive forces of the periodic structure, whereas the pairwise approach in *CrystalExplorer* focuses on the partitioning of energies into discrete molecular contacts [40, 52].

As shown in Table 4, both methods confirm that the 1:1:1 cocrystal hydrate is an energetically robust phase. The substantial stabilization compared to the pure CAF lattice provides a quantitative basis for the stability of this stoichiometric arrangement. These findings support the theoretical prediction that the 1:1:1 ratio is thermodynamically favorable in the presence of lattice water, which acts as a “supramolecular glue” to satisfy the hydrogen‐bonding requirements of both the phenol and carbonyl groups [25, 29].

**TABLE 4:** Comparison of lattice energies calculated using *CrystalExplorer21* (CE) and CP2K.

Method	Functional and basis set	Lattice energy, kJ/mol
CE	B3LYP/6‐31G(d,p)	−51.2
CP2K	DFT‐D3(BJ)	−77.26

**FIGURE 6:**
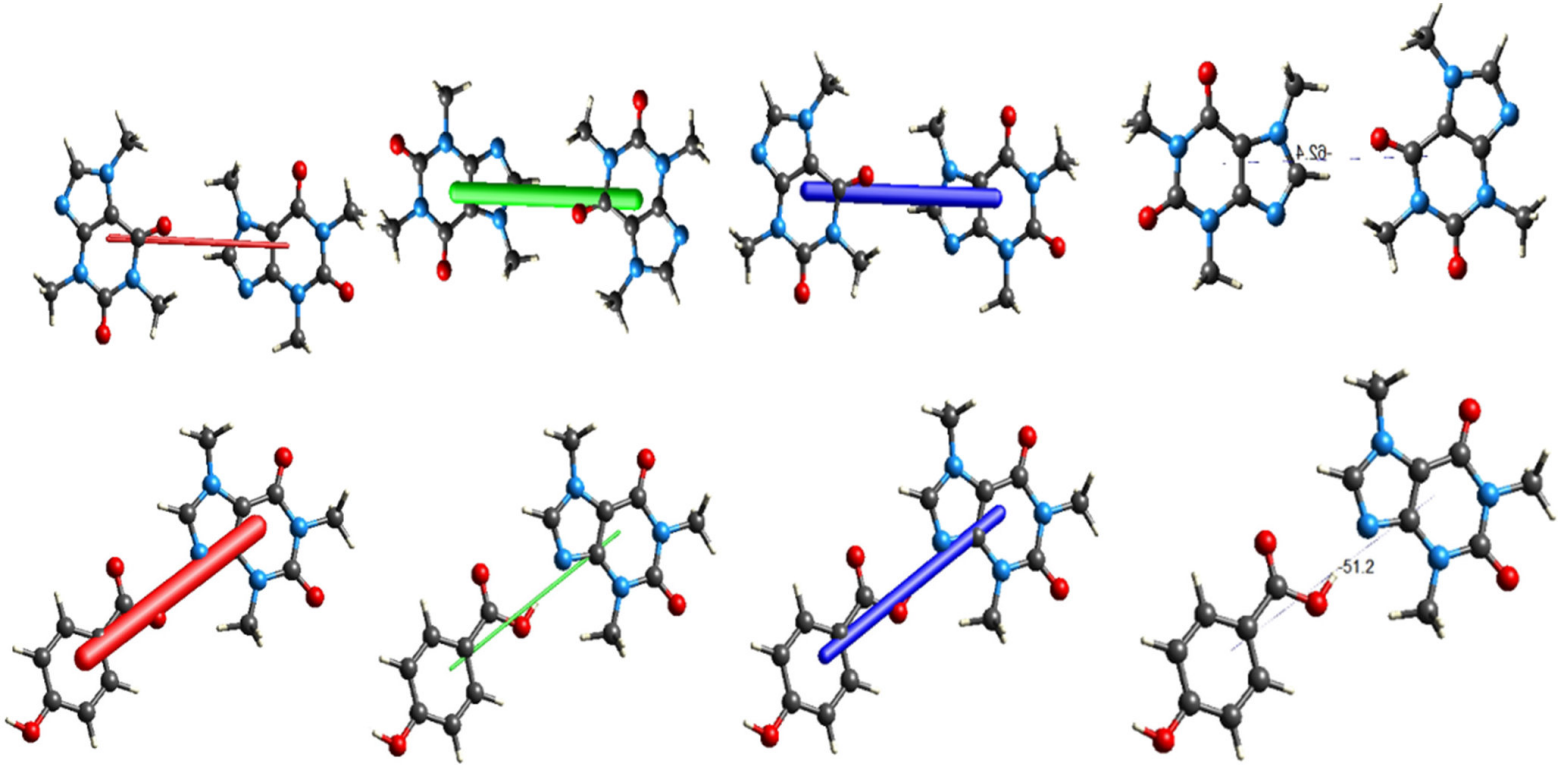
Energy frameworks for CAF and CAF·4HBA·H_2_O, the electrostatic (red) and dispersion (green) components, and the total interaction energy (blue), with the total interaction energy being annotated. Cylinder radii are proportional to the corresponding energy; a scale factor of 50 and a cut‐off value of 10 kJ/mol have been used.

Figure 6 provides energy frameworks for the crystals, where the electrostatic (red), dispersion (green), and total interaction energy (blue or yellow) components are visualized. These figures help identify the directional nature of specific interactions in the lattice, particularly the electrostatic forces, which often appear in consistent directions within the lattice. Such patterns can be used to identify key interactions driving the stability of the cocrystal hydrate.

A detailed breakdown of the intermolecular interactions stabilizing CAF and CAF·4HBA·H_2_O was performed using energy decomposition analysis, with the individual terms calculated, scaled, and then summed to obtain the total energy.

Energy framework analysis provides quantitative insights into the nature and strength of intermolecular interactions within the CAF and CAF·4HBA·H_2_O structure, as summarized in Table S10, which lists all calculated pairwise interaction energies, corresponding molecular pairs, centroid distances, and symmetry‐related contacts derived from the CE‐B3LYP (B3LYP/6‐31G(d,p)) model.

For CAF, the most stabilizing interaction is observed between CAF molecules related by the symmetry operation (*x*, –*y* + ½, *z* + ½) and corresponds to a glide‐related neighbor generated by a translation along the *c* axis combined with inversion of the y coordinate. This CAF···CAF contact occurs at a centroid distance of 7.63 Å and shows a total interaction energy (*E*_tot_) of −17.7 kJ/mol, dominated by electrostatic (*E*_ele_ = −11.4 kJ/mol) and dispersion (*E*_dis_ = −6.8 kJ/mol) contributions, indicating a balance of weak electrostatic complementarity and π···π /van der Waals interactions.

A second key stabilizing CAF···CAF interaction corresponds to molecules related by the symmetry operation (−*x*, −*y*, −*z*) and represents an inversion‐related pair of CAF molecules forming an antiparallel arrangement in the crystal packing. This interaction at a centroid distance of 7.68 Å yields an *E*_tot_ of −14.5 kJ/mol, primarily stabilized by electrostatic and dispersion contributions, consistent with aromatic stacking and shape complementarity between inversion‐related CAF dimers.

In contrast, the interaction at 3.97 Å corresponds to a close‐contact CAF···CAF interaction between translationally related molecules within the same stacking motif (*x*, *y*, *z*). Despite a strong dispersion contribution (*E*_dis_ = −41.7 kJ/mol), this contact exhibits significant electrostatic repulsion (*E*_ele_ = 28.1 kJ/mol) and short‐range steric repulsion (*E*_rep_ = 14.0 kJ/mol), resulting in only weak net stabilization (*E*_tot_ = −4.6 kJ/mol). This indicates that the interaction is geometry‐constrained rather than strongly directional.

For CAF·4HBA·H_2_O, the strongest interaction corresponds to a hydrogen‐bonding assembly, where water acts as a bridging unit connecting CAF heteroatom sites with the hydroxyl and carboxyl groups of 4HBA. This interaction occurs at a centroid distance of 6.61 Å and exhibits a markedly stabilizing *E*_tot_ of −51.2 kJ/‌mol, driven by strong electrostatic (*E*_ele_ = −78.8 kJ/mol) and polarization (*E*_pol_ = −17.1 kJ/mol) contributions, partially offset by repulsion (*E*_rep_ = 94.1 kJ/mol). The magnitude of this interaction reflects the formation of a cooperative hydrogen‐bonded network involving both 4HBA and lattice water.

These results are consistent with the scale factors used for energy decomposition, as outlined in Mackenzie *et al.* (2017) [53], which use *k*_ele_, *k*_pol_, *k*_disp_, and *k*_rep_ values of 1.057, 0.740, 0.871, and 0.618 for the CE‐B3LYP (B3LYP/6‐31G(d,p) electron densities) model (Table S9). These scale factors help quantify the relative contributions of each energy term to the overall lattice energy.

### 3.8 Solubility Studies

The solubility profile of CAF, 4‐hydroxybenzoic acid (4HBA), and their cocrystal hydrate demonstrates notable differences under simulated gastric (pH 1.2) and intestinal (pH 6.8) conditions as summarized in Table 5. Pure CAF exhibited high aqueous solubility, with values of 27.1 mg/mL at pH 1.2 and 21.7 mg/mL at pH 6.8. In contrast, the CAF·4HBA·H_2_O showed significantly lower solubility, reaching 19.1 mg/mL in acidic media and 10.4 mg/mL in phosphate buffer, indicating a pH‐dependent decrease in solubility compared to pure CAF at both pH levels. While 4HBA alone displayed lower solubility than CAF (5.2 mg/mL at pH 1.2 and 5 mg/mL at pH 6.8), its contribution to overall solubility was limited.

**TABLE 5:** Solubility of CAF, 4HBA, and CAF·4HBA·H_2_O under simulated gastric (pH 1.2) and intestinal (pH 6.8) conditions.

Sample	Solubility at pH 1.2, mg/mL	Solubility at pH 6.8, mg/mL
CAF	27.1	21.7
4HBA	5.2	5.0
CAF·4HBA·H_2_O	19.1	10.4

Furthermore, the physical mixture of CAF and 4HBA yielded only marginal changes compared to individual components, indicating that mechanical blending does not sufficiently disrupt the crystalline structure to improve dissolution. The changed solubility behavior of the cocrystal hydrate can be explained by a change in intermolecular interactions in the crystal lattice that affect the dissolution kinetics and phase behavior upon exposure to varying pH conditions. The release behavior of CAF is modulated only by partial recrystallization or dissociation during dissolution, although its intrinsic solubility of CAF is already high. These findings indicate that cocrystallization in this system is used to optimize dissolution properties rather than to improve the inherent aqueous solubility of CAF as evidenced by the solubility values presented in Table 5.

### 3.9 Gastrointestinal Release Profile

**FIGURE 7:**
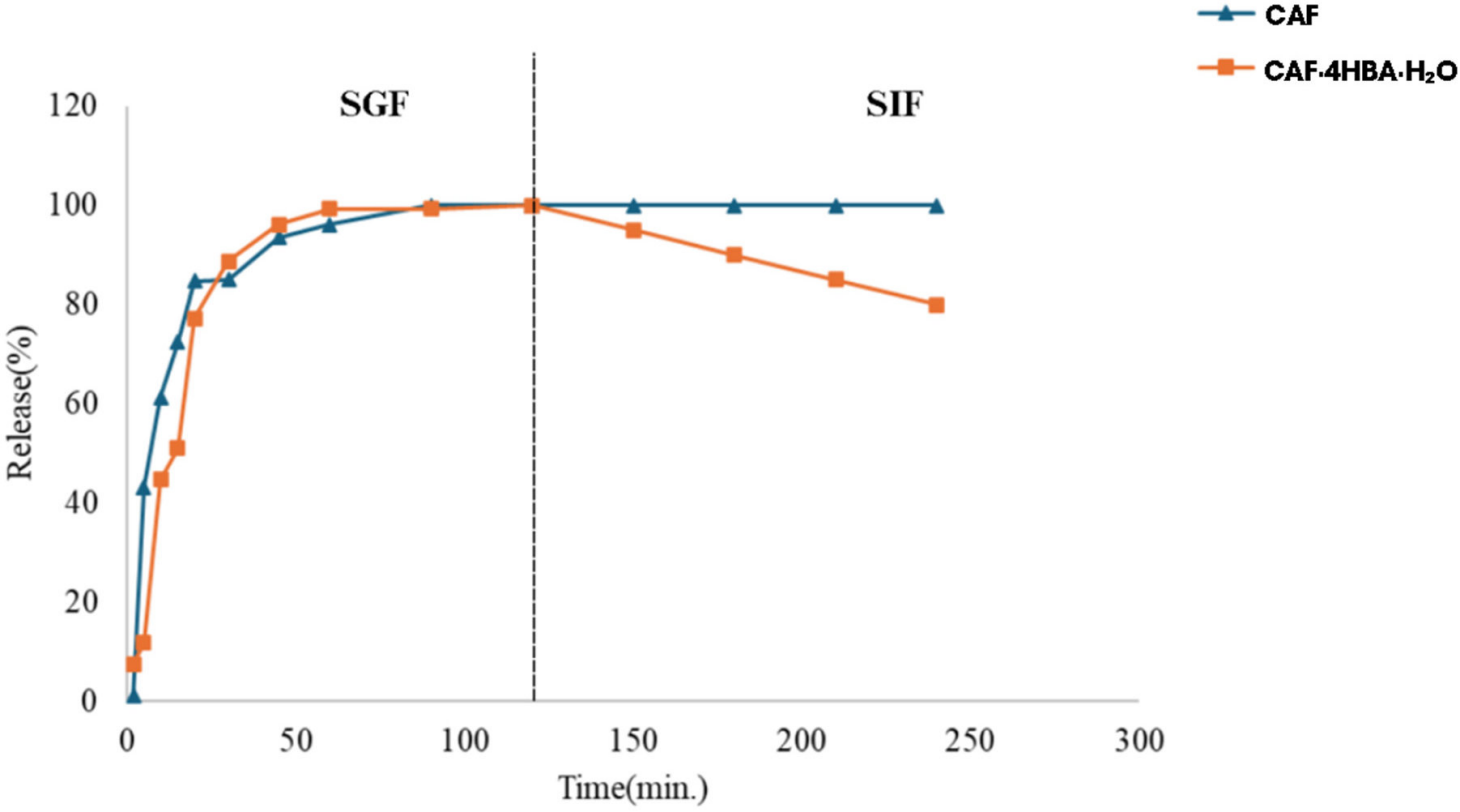
*In vitro* release profile of caffeine (CAF) from free CAF and CAF·4HBA·H_2_O under simulated gastrointestinal conditions. The digestion was performed sequentially in simulated gastric fluid (0–120 min) and simulated intestinal fluid (120–240 min). Data represent mean ± standard deviation (*n* = 3).

The release profiles of pure CAF and CAF from the CAF·4HBA·H_2_O during the simulated gastrointestinal digestion experiment are shown in Figure 7. In the simulated gastric fluid (SGF, 0.01 N HCl, pH 1.2), the cocrystal hydrate released only 2.8% of CAF initially, gradually increasing to about 89.2% by 60 min and nearly complete release by 120 min. In contrast, pure CAF showed a faster initial release of 7.5% at 2 min but reached complete release by 120 min, like the cocrystal hydrate. Upon transition to SIF (PBS, pH 6.8), the CAF·4HBA·H_2_O exhibited rapid and sustained release, achieving nearly 100% release by 60 min. Pure CAF, however, showed a slower dissolution rate, reaching 93.5% release at the same time point.

Comparing both phases, the cocrystal hydrate demonstrated a slower and more controlled release in SGF but a faster and more complete release in SIF compared to pure CAF. This contrast suggests that the cocrystal hydrate moderates CAF release in the gastric environment, potentially reducing gastric irritation, while enhancing dissolution and bioavailability in the intestinal environment. These findings suggest that the CAF cocrystal hydrate may provide better modified release behavior and enhanced dissolution performance in intestinal conditions, particularly in physiological pH conditions.

## 4 Conclusion

This study successfully demonstrates the formation of a cocrystal hydrate comprising CAF and 4‐hydroxybenzoic acid (4HBA), confirmed through single‐crystal X‐ray diffraction and supported by thermal, spectroscopic, and computational analyses. The cocrystal hydrate crystallized in the triclinic *P‐1* space group and exhibited a distinct melting point significantly lower than either parent compound, indicating the formation of a new solid phase. Hirshfeld surface analysis and energy framework calculations revealed substantial alterations in intermolecular interactions, particularly enhanced hydrogen bonding and dispersion forces, contributing to the stability of the new crystal lattice. Moreover, solubility and *in vitro* digestion studies revealed enhanced dissolution of CAF upon cocrystallization, indicating altered dissolution behavior and potential modulation of absorption kinetics. Together, these findings support the utility of cocrystallization as a viable strategy for modulating the physicochemical properties of APIs and lay the groundwork for further pharmaceutical development of CAF·4HBA·H_2_O system.

## Conflicts of Interest

The authors declare no conflicts of interest.

## Supporting information

Tables of crystal data, atomic positions and thermal parameters, anisotropic thermal parameters, bond distances, and bond angles have been deposited as CIF. CCDC Deposition Number 2563394.

## Data Availability

The data that support the findings of this study are available in the Supporting Information of this article.
