# Supplementary material for: Caffeine and 4‐Hydroxybenzoic Acid Cocrystal Hydrate: Preparation, Characterization, and Evaluation
Source: Chemphyschem. 2026 Aug 25;27(16):e70545. doi: 10.1002/cphc.70545 (PMC13506356; doi:10.1002/cphc.70545)
Supplement: Supplementary file 1 [file CPHC-27-e70545-s001.pdf]

Caffeine and 4-Hydroxybenzoic Acid Cocrystal Hydrate: Preparation, Characterization, and Evaluation

Shagun Gogna[b], Jonathan J. Du[b], Paul W. Groundwater[b], Mohamed Fares[b], Jan-Willem C. Alffenaar[b], David E. Hibbs *[a]

[a] David E. Hibbs*
Sydney Pharmacy School, Faculty of Medicine and Health
The University of Sydney
Pharmacy and Bank Building, Camperdown, NSW 2006, Australia
E-mail: david.hibbs@sydney.edu.au

[b] Shagun Gogna[1,2,3], Jonathan J. Du[1], Paul W. Groundwater[1], Mohamed Fares[1], Jan-Willem C. Alffenaar[1,2,3]
[1] Sydney Pharmacy School, Faculty of Medicine and Health, The University of Sydney, Camperdown, NSW, Australia
[2] Sydney Institute for Infectious Diseases, The University of Sydney, Sydney, NSW, Australia
[3] Westmead Hospital, Sydney, NSW, Australia

Table S1. Crystallographic details for CAF·4HBA·H_2O

	**CAF·4HBA·H_2O**
Formula	$C_{15}H_{16}N_4O_5.H_2O$
Molecular mass (g/mol)	350.33
Crystal size (mm)	0.10 × 0.09 × 0.08
Temperature (K)	150(2)
Crystal system	Triclinic
Space group	P -1
a (Å)	7.2413(2)
b (Å)	8.5339(2)
c (Å)	13.8568(3)
α (°)	90.264(2)
β (°)	101.385(2)
γ (°)	107.608(2)
Volume ($Å^3$)	798.22(4)
Z, Calculated density (Mg/m^3)	2, 1.458
Refinement method	Full-matrix least-squares on F^2
No. of reflections collected	7995
No. unique	3036
R_{int}	0.0204
Completeness (%)	99.2
No. reflections used	3036
ρ_c (g/cm)	1.458

$F(000)$	368
μ (m/m)	0.971
$\sin\theta/\lambda_{max}$	0.621
Index ranges	$-8\leq h\leq 8$,<br>$-10\leq k\leq 10$,<br>$-17\leq l\leq 16$
Data/restraints/parameters	3036 / 2 / 298
GOF	1.032
Final R indexes($I \geq 2\sigma(I)$)	R1 = 0.0340,<br>wR2 = 0.0921
Final R indexes (all data)	R1 = 0.0358,<br>wR2 = 0.0935
Largest diff. peak/hole (e/Å^3)	0.240 and -0.257

Table S2. Atomic coordinates (x 10^4) and equivalent isotropic displacement parameters (Å^{2}x 10^3) for CAF·4HBA·H_2O. U(eq) is defined as one third of the trace of the orthogonalized Uij tensor.

**Atom**	**x**	**y**	**z**	**U(eq)**
C(1)	6674(2)	4502(1)	8944(1)	22(1)
C(2)	4707(2)	3479(1)	7359(1)	19(1)
C(3)	4510(2)	1888(1)	7589(1)	19(1)
C(4)	5509(2)	1481(1)	8499(1)	19(1)
C(5)	2905(2)	1976(2)	6098(1)	23(1)
C(6)	5982(2)	6490(2)	7764(1)	34(1)
C(7)	7572(2)	2603(2)	10121(1)	31(1)
C(8)	2576(2)	-876(2)	6654(1)	28(1)
C(9)	1572(2)	6550(2)	3701(1)	22(1)
C(10)	361(2)	6019(2)	2768(1)	23(1)
C(11)	-141(2)	7133(2)	2125(1)	23(1)
C(12)	557(2)	8818(2)	2411(1)	22(1)
C(13)	1784(2)	9363(2)	3340(1)	24(1)
C(14)	2284(2)	8237(2)	3974(1)	23(1)
C(15)	2057(2)	5308(2)	4369(1)	24(1)
N(1)	6541(1)	2877(1)	9141(1)	21(1)
N(2)	5787(2)	4791(1)	8020(1)	22(1)
N(3)	3326(1)	924(1)	6759(1)	21(1)
N(4)	3726(2)	3552(1)	6428(1)	23(1)
O(1)	5531(1)	97(1)	8729(1)	24(1)
O(2)	7582(1)	5631(1)	9572(1)	33(1)
O(4)	3084(2)	5954(1)	5262(1)	31(1)
O(3)	1567(1)	3845(1)	4129(1)	34(1)
O(5)	99(1)	9967(1)	1828(1)	28(1)
O(6)	-2418(1)	8647(1)	184(1)	32(1)

Table S3. Bond lengths [Å] and angles [°] for CAF·4HBA·H_2O.

Bond	Bond Length [Å]	Angle	Bond Angle [°]
C(1)-O(2)	1.2254(15)	O(2)-C(1)-N(2)	121.49(11)
C(1)-N(2)	1.3740(15)	O(2)-C(1)-N(1)	120.77(11)
C(1)-N(1)	1.3921(15)	N(2)-C(1)-N(1)	117.73(10)
C(2)-N(4)	1.3559(15)	N(4)-C(2)-C(3)	111.65(10)
C(2)-C(3)	1.3678(16)	N(4)-C(2)-N(2)	126.55(10)
C(2)-N(2)	1.3720(15)	C(3)-C(2)-N(2)	121.80(10)
C(3)-N(3)	1.3819(15)	C(2)-C(3)-N(3)	105.35(10)
C(3)-C(4)	1.4249(16)	C(2)-C(3)-C(4)	122.64(11)
C(4)-O(1)	1.2287(14)	N(3)-C(3)-C(4)	131.88(10)
C(4)-N(1)	1.3993(15)	O(1)-C(4)-N(1)	121.48(10)
C(5)-N(4)	1.3319(16)	O(1)-C(4)-C(3)	126.49(11)
C(5)-N(3)	1.3424(15)	N(1)-C(4)-C(3)	112.03(10)
C(5)-H(5)	0.967(16)	N(4)-C(5)-N(3)	113.39(10)
C(6)-N(2)	1.4657(15)	N(4)-C(5)-H(5)	125.0(9)
C(6)-H(6A)	0.98(2)	N(3)-C(5)-H(5)	121.6(9)
C(6)-H(6B)	0.95(2)	N(2)-C(6)-H(6A)	109.6(12)
C(6)-H(6C)	1.01(2)	N(2)-C(6)-H(6B)	109.2(13)
C(7)-N(1)	1.4704(15)	H(6A)-C(6)-H(6B)	108.3(17)
C(7)-H(7A)	0.952(19)	N(2)-C(6)-H(6C)	110.8(13)
C(7)-H(7B)	1.00(2)	H(6A)-C(6)-H(6C)	109.5(17)
C(7)-H(7C)	0.92(2)	H(6B)-C(6)-H(6C)	109.4(18)
C(8)-N(3)	1.4617(15)	N(1)-C(7)-H(7A)	109.5(11)
C(8)-H(8A)	0.967(19)	N(1)-C(7)-H(7B)	110.2(14)
C(8)-H(8B)	0.97(2)	H(7A)-C(7)-H(7B)	106.1(17)
C(8)-H(8C)	0.966(19)	N(1)-C(7)-H(7C)	110.8(15)
C(9)-C(10)	1.3964(17)	H(7A)-C(7)-H(7C)	110.6(18)
C(9)-C(14)	1.3966(17)	H(7B)-C(7)-H(7C)	110(2)
C(9)-C(15)	1.4868(16)	N(3)-C(8)-H(8A)	109.8(11)
C(10)-C(11)	1.3856(17)	N(3)-C(8)-H(8B)	108.4(11)
C(10)-H(10)	0.978(17)	H(8A)-C(8)-H(8B)	107.5(15)
C(11)-C(12)	1.3987(17)	N(3)-C(8)-H(8C)	109.1(11)
C(11)-H(11)	0.954(16)	H(8A)-C(8)-H(8C)	112.4(15)
C(12)-O(5)	1.3502(14)	H(8B)-C(8)-H(8C)	109.5(15)
C(12)-C(13)	1.3986(17)	C(10)-C(9)-C(14)	118.67(11)
C(13)-C(14)	1.3854(17)	C(10)-C(9)-C(15)	119.16(11)
C(13)-H(13)	0.979(16)	C(14)-C(9)-C(15)	122.17(11)
C(14)-H(14)	0.999(16)	C(11)-C(10)-C(9)	121.05(11)
C(15)-O(3)	1.2155(16)	C(11)-C(10)-H(10)	120.3(10)
C(15)-O(4)	1.3301(15)	C(9)-C(10)-H(10)	118.7(10)
O(4)-H(4)	0.97(2)	C(10)-C(11)-C(12)	119.86(11)
O(5)-H(5A)	0.96(2)	C(10)-C(11)-H(11)	118.4(10)
O(6)-H(6D)	0.9598(11)	C(12)-C(11)-H(11)	121.8(10)
O(6)-H(6E)	0.9598(11)	O(5)-C(12)-C(13)	117.58(11)
		O(5)-C(12)-C(11)	122.93(11)
		C(13)-C(12)-C(11)	119.49(11)
		C(14)-C(13)-C(12)	120.07(11)

		C(14)-C(13)-H(13)	121.2(9)
		C(12)-C(13)-H(13)	118.7(9)
		C(13)-C(14)-C(9)	120.85(11)
		C(13)-C(14)-H(14)	119.3(9)
		C(9)-C(14)-H(14)	119.8(9)
		O(3)-C(15)-O(4)	123.02(11)
		O(3)-C(15)-C(9)	123.62(11)
		O(4)-C(15)-C(9)	113.36(10)
		C(1)-N(1)-C(4)	126.26(10)
		C(1)-N(1)-C(7)	116.81(10)
		C(4)-N(1)-C(7)	116.93(10)
		C(2)-N(2)-C(1)	119.25(10)
		C(2)-N(2)-C(6)	121.26(10)
		C(1)-N(2)-C(6)	119.49(10)
		C(5)-N(3)-C(3)	105.95(9)
		C(5)-N(3)-C(8)	127.22(11)
		C(3)-N(3)-C(8)	126.70(10)
		C(5)-N(4)-C(2)	103.65(10)
		C(15)-O(4)-H(4)	108.2(13)
		C(12)-O(5)-H(5A)	108.8(12)
		H(6D)-O(6)-H(6E)	106.8(16)

Table S4. Anisotropic displacement parameters (Å^{2}x 10^3) for CAF·4HBA·H_2O. The anisotropic displacement factor exponent takes the form: $-2\pi^2$ [$h^2 a^{*2}U^{11}$ + ... + 2 h k a* b* U^{12}].

**Atom**	**U11**	**U22**	**U33**	**U23**	**U13**	**U12**
C(1)	24(1)	19(1)	23(1)	2(1)	3(1)	6(1)
C(2)	20(1)	20(1)	19(1)	4(1)	5(1)	7(1)
C(3)	20(1)	18(1)	19(1)	2(1)	4(1)	6(1)
C(4)	19(1)	18(1)	19(1)	3(1)	5(1)	5(1)
C(5)	23(1)	28(1)	18(1)	3(1)	2(1)	9(1)
C(6)	50(1)	18(1)	32(1)	6(1)	0(1)	10(1)
C(7)	37(1)	25(1)	21(1)	5(1)	-6(1)	4(1)
C(8)	35(1)	20(1)	25(1)	-2(1)	2(1)	5(1)
C(9)	21(1)	26(1)	20(1)	6(1)	6(1)	9(1)
C(10)	23(1)	23(1)	23(1)	3(1)	4(1)	7(1)
C(11)	22(1)	27(1)	19(1)	3(1)	2(1)	7(1)
C(12)	23(1)	25(1)	20(1)	7(1)	7(1)	9(1)
C(13)	26(1)	24(1)	22(1)	3(1)	5(1)	7(1)
C(14)	24(1)	28(1)	18(1)	3(1)	4(1)	8(1)
C(15)	23(1)	27(1)	23(1)	5(1)	5(1)	8(1)
N(1)	24(1)	18(1)	19(1)	3(1)	1(1)	5(1)
N(2)	27(1)	15(1)	22(1)	3(1)	3(1)	6(1)
N(3)	22(1)	21(1)	18(1)	0(1)	2(1)	6(1)
N(4)	24(1)	25(1)	19(1)	5(1)	3(1)	10(1)
O(1)	30(1)	18(1)	23(1)	5(1)	3(1)	9(1)
O(2)	42(1)	20(1)	29(1)	-3(1)	-7(1)	7(1)

O(4)	46(1)	28(1)	21(1)	5(1)	-1(1)	17(1)
O(3)	39(1)	24(1)	32(1)	6(1)	-2(1)	8(1)
O(5)	35(1)	25(1)	23(1)	7(1)	0(1)	10(1)
O(6)	44(1)	29(1)	23(1)	0(1)	-4(1)	20(1)

Table S5. Hydrogen coordinates (x 10^4) and isotropic displacement parameters ($Å^2$x 10^3) for CAF·4HBA·H_2O.

**Atom**	**x**	**y**	**z**	**U(eq)**
H(14)	3190(20)	8643(19)	4629(12)	28(4)
H(5)	2080(20)	1592(18)	5451(11)	25(4)
H(10)	-120(20)	4840(20)	2572(12)	31(4)
H(13)	2270(20)	10550(20)	3529(11)	29(4)
H(11)	-990(20)	6716(19)	1499(12)	29(4)
H(7A)	6720(30)	1710(20)	10389(13)	41(4)
H(8A)	3630(30)	-1320(20)	6590(14)	46(5)
H(6D)	-2500(30)	7600(11)	-108(13)	54(5)
H(8B)	1570(30)	-1210(20)	6047(15)	50(5)
H(8C)	1990(30)	-1270(20)	7210(14)	46(5)
H(6E)	-2930(30)	9240(20)	-338(9)	54(5)
H(5A)	-780(30)	9420(30)	1228(16)	57(6)
H(6A)	7330(30)	7200(30)	8049(15)	57(5)
H(6B)	5770(30)	6530(30)	7068(17)	61(6)
H(7B)	8750(40)	2260(30)	10062(17)	75(7)
H(7C)	7970(30)	3540(30)	10537(18)	74(7)
H(4)	3360(30)	5070(30)	5646(16)	63(6)
H(6C)	4990(30)	6910(30)	8020(16)	68(6)

Table S6. Torsion angles [°]for CAF·4HBA·H_2O.

**Atoms**	**Angle [°]**
N(4)-C(2)-C(3)-N(3)	-0.63(13)
N(2)-C(2)-C(3)-N(3)	179.39(10)
N(4)-C(2)-C(3)-C(4)	175.72(10)
N(2)-C(2)-C(3)-C(4)	-4.26(17)
C(2)-C(3)-C(4)-O(1)	-173.95(11)
N(3)-C(3)-C(4)-O(1)	1.3(2)
C(2)-C(3)-C(4)-N(1)	5.24(16)
N(3)-C(3)-C(4)-N(1)	-179.49(11)
C(14)-C(9)-C(10)-C(11)	-0.41(17)
C(15)-C(9)-C(10)-C(11)	179.45(10)
C(9)-C(10)-C(11)-C(12)	-0.44(18)
C(10)-C(11)-C(12)-O(5)	-178.91(11)
C(10)-C(11)-C(12)-C(13)	0.98(17)
O(5)-C(12)-C(13)-C(14)	179.24(10)
C(11)-C(12)-C(13)-C(14)	-0.66(18)
C(12)-C(13)-C(14)-C(9)	-0.20(18)
C(10)-C(9)-C(14)-C(13)	0.73(17)
C(15)-C(9)-C(14)-C(13)	-179.12(11)

C(10)-C(9)-C(15)-O(3)	5.08(18)
C(14)-C(9)-C(15)-O(3)	-175.07(11)
C(10)-C(9)-C(15)-O(4)	-174.87(10)
C(14)-C(9)-C(15)-O(4)	4.99(16)
O(2)-C(1)-N(1)-C(4)	178.13(11)
N(2)-C(1)-N(1)-C(4)	-2.99(18)
O(2)-C(1)-N(1)-C(7)	-1.36(17)
N(2)-C(1)-N(1)-C(7)	177.52(11)
O(1)-C(4)-N(1)-C(1)	177.57(10)
C(3)-C(4)-N(1)-C(1)	-1.67(16)
O(1)-C(4)-N(1)-C(7)	-2.94(17)
C(3)-C(4)-N(1)-C(7)	177.82(10)
N(4)-C(2)-N(2)-C(1)	179.15(11)
C(3)-C(2)-N(2)-C(1)	-0.88(17)
N(4)-C(2)-N(2)-C(6)	0.16(18)
C(3)-C(2)-N(2)-C(6)	-179.87(11)
O(2)-C(1)-N(2)-C(2)	-176.86(11)
N(1)-C(1)-N(2)-C(2)	4.27(16)
O(2)-C(1)-N(2)-C(6)	2.14(18)
N(1)-C(1)-N(2)-C(6)	-176.72(11)
N(4)-C(5)-N(3)-C(3)	0.22(13)
N(4)-C(5)-N(3)-C(8)	176.32(11)
C(2)-C(3)-N(3)-C(5)	0.25(12)
C(4)-C(3)-N(3)-C(5)	-175.63(12)
C(2)-C(3)-N(3)-C(8)	-175.87(11)
C(4)-C(3)-N(3)-C(8)	8.3(2)
N(3)-C(5)-N(4)-C(2)	-0.59(13)
C(3)-C(2)-N(4)-C(5)	0.75(13)
N(2)-C(2)-N(4)-C(5)	-179.28(11)

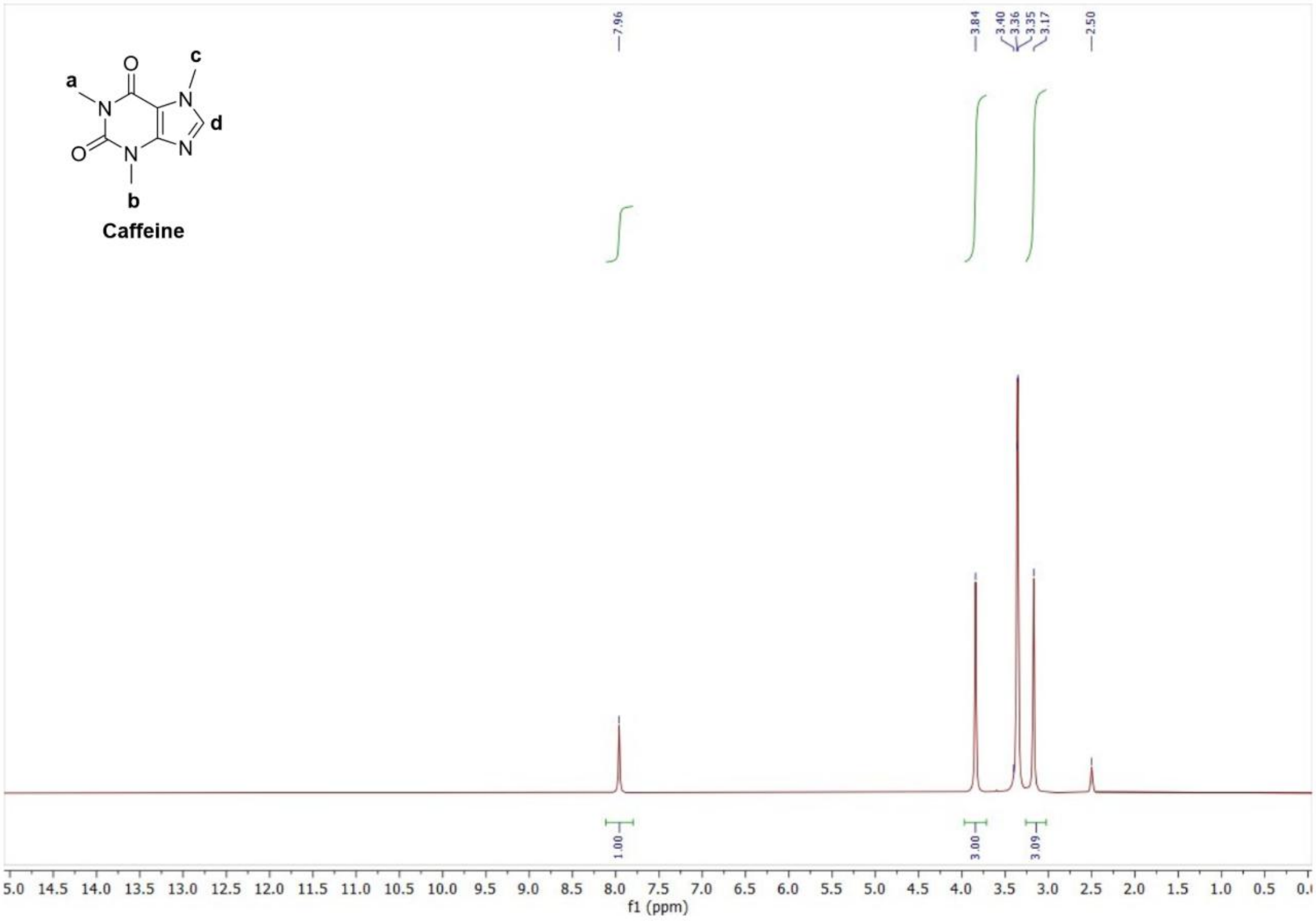

Caffeine
a
b
c
d
7.96
3.84
3.40
3.36
3.35
3.17
2.50
1.00
3.00
3.09
f1 (ppm)

(a)

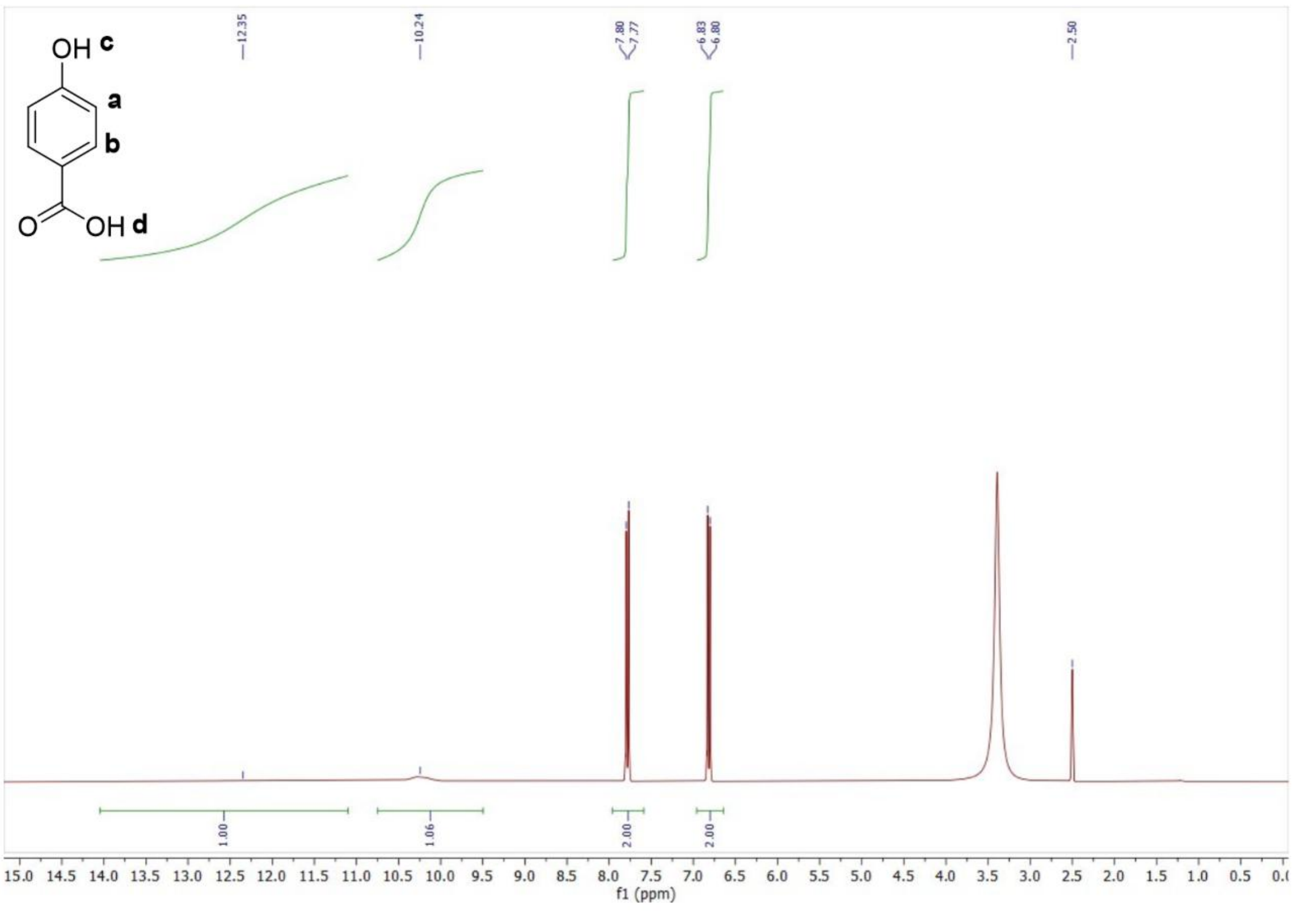

OH c
a
b
OH d
12.35
10.24
7.80
7.77
6.83
6.80
2.50
1.00
1.06
2.00
2.00
f1 (ppm)

(b)

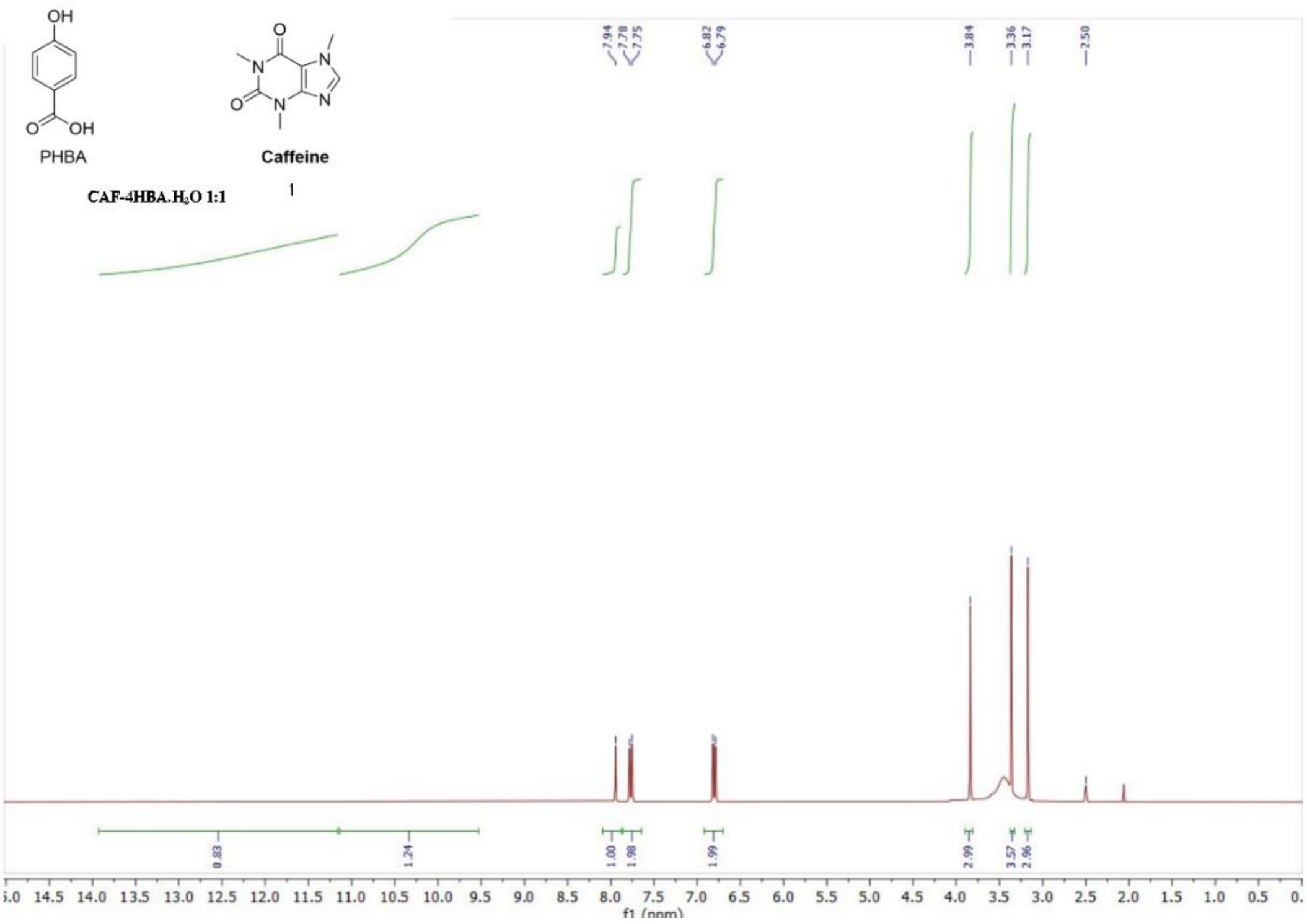

(c)

Figure S1. ^{1}H NMR of (a) CAF, (b) 4-HBA and (c) CAF·4HBA·H_2O.

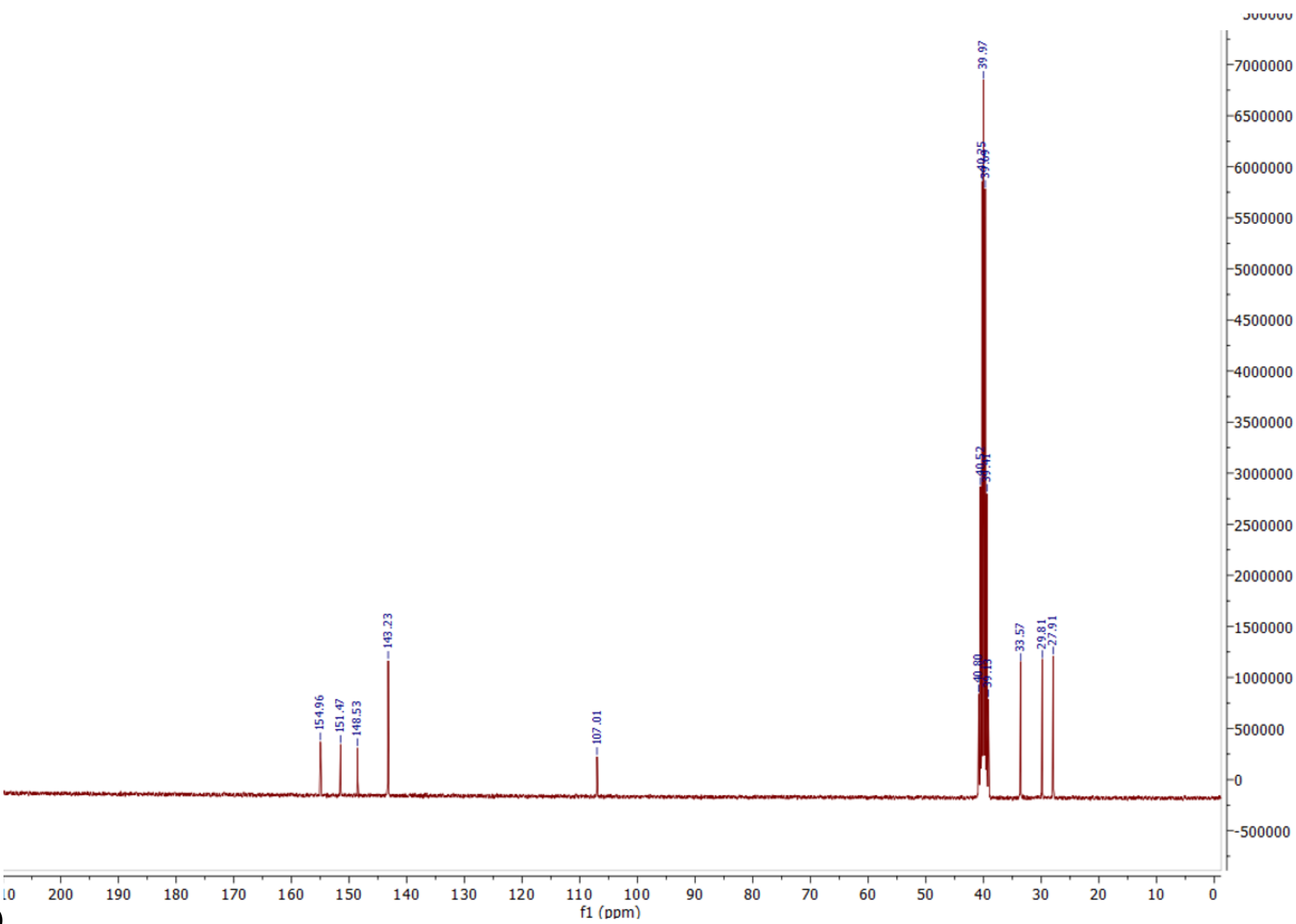

(a)

(b)

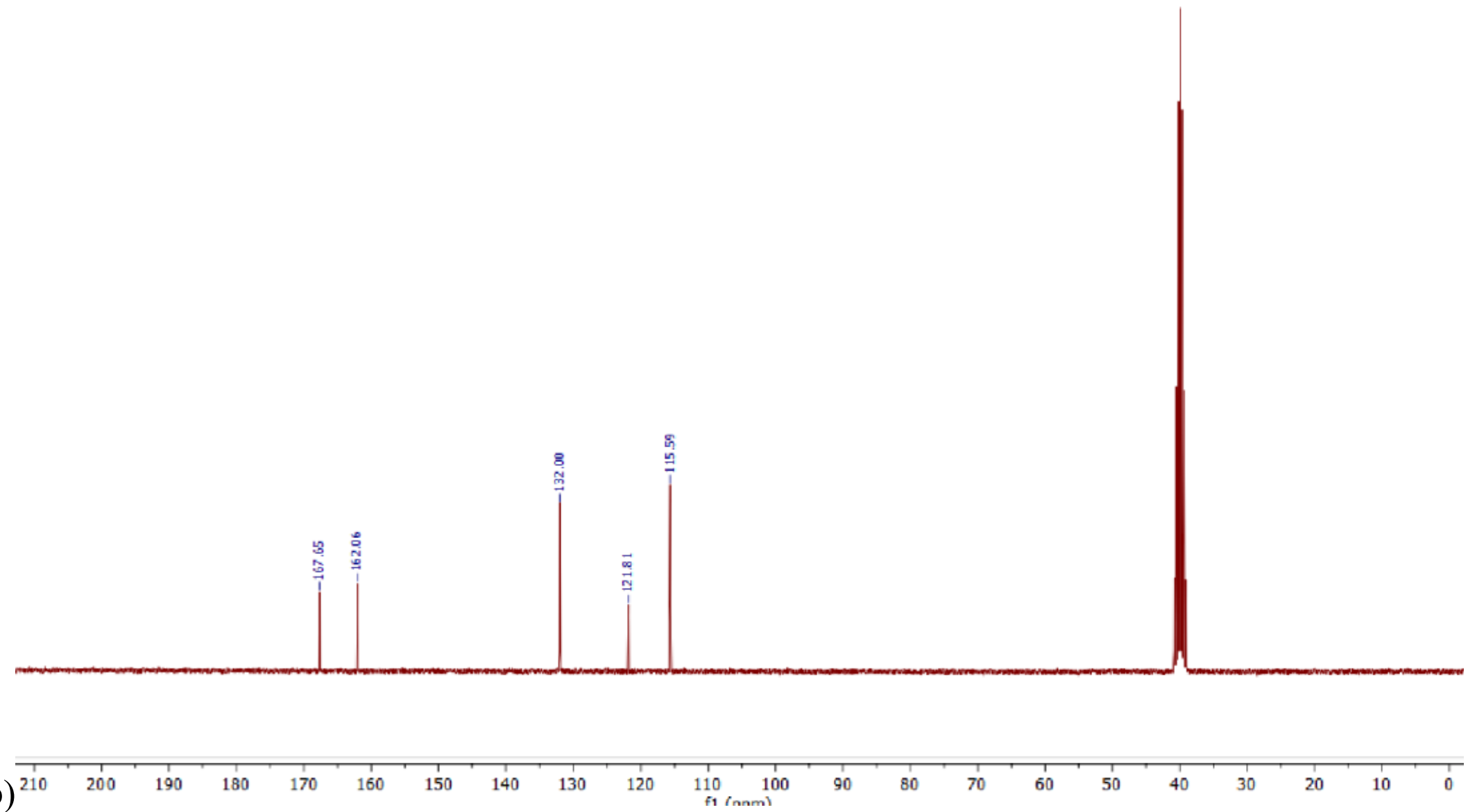

(c)

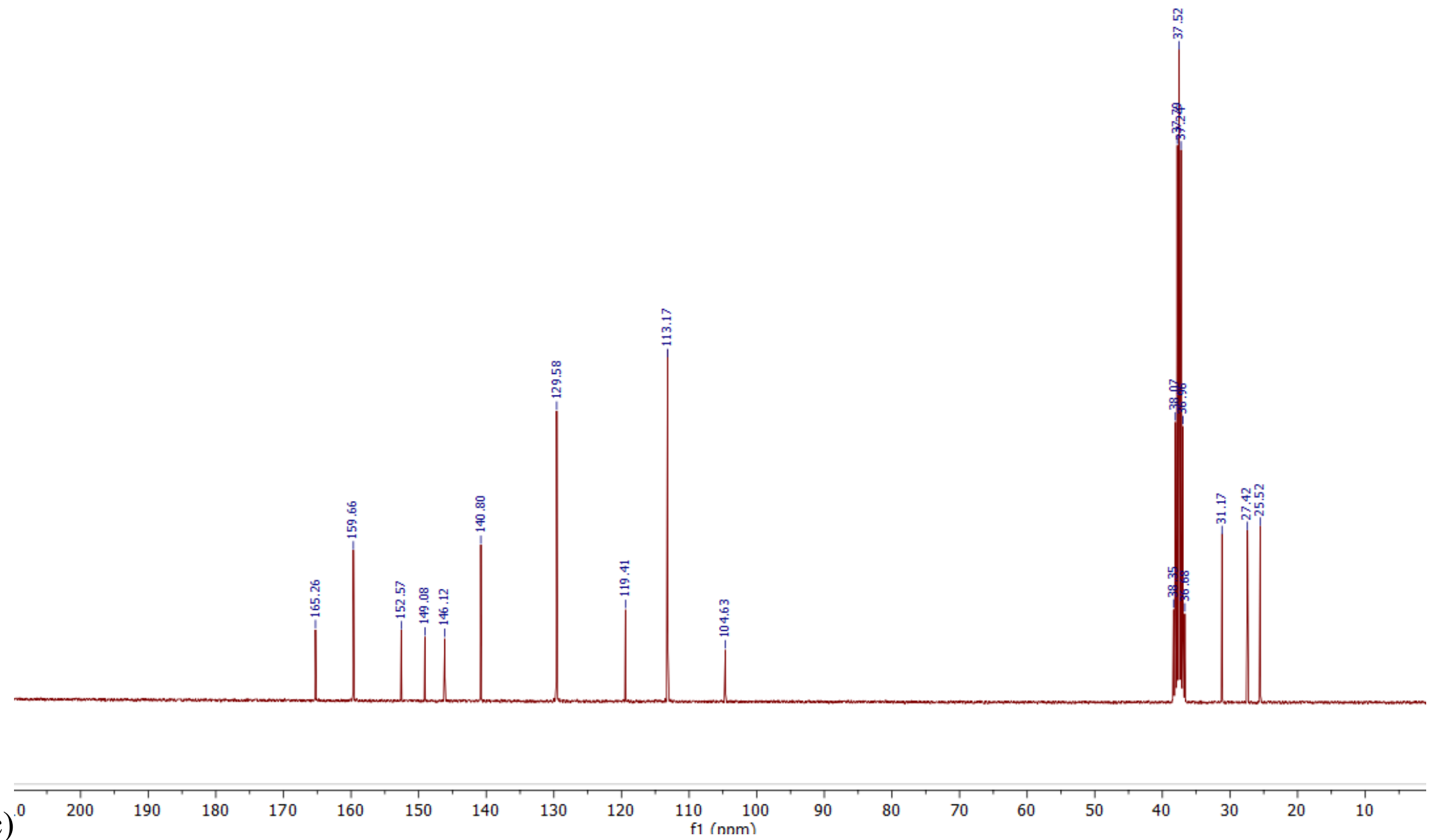

Figure S2. ^{13}C NMR of (a) CAF, (b) 4-HBA and (c) CAF·4HBA·H_2O.

Table S7. Hirshfield statistics for CAF·4HBA·H_2O.

Parameter	CAF	CAF·4HBA·H_2O
Surface Volume (Å³)	220.99	368.00
Surface Area (Å³)	221.61	355.84
Globularity	0.798	0.698
Asphericity	0.121	0.366

Mean d_i (Å^3)	1.6320	1.6256
Mean d^e (Å^3)	1.6642	1.6381
d_{norm}	0.4677	0.4194
Shape Index	0.2937	0.2476
Curvedness	-1.0156	-0.9977

Table S8. 2D Fingerprint Plot Contributions for CAF·4HBA·H_2O.

Contact Type	CAF	CAF·4HBA·H_2O
H···H	45.5%	45.5%
H···O / O···H	10.3%	12.1 %
H···C / C···H	2.1%	3.3%
H···N / N···H	6.8%	1.0%
C···O / O···C	0.1%	1.5%
N···C / C···N	3.5%	2.6%
C···C	4.5%	6.3%
Total H-involving Contacts	66.9%	66.9%

Table S9. Scale Factors for Benchmarked Energy Models

Energy Model	k_{ele}	k_{pol}	k_{disp}	k_{rep}
CE-HF (HF/3-21G electron densities)	1.019	0.651	0.901	0.811
CE-B3LYP (B3LYP/6-31G(d,p) electron densities)	1.057	0.740	0.871	0.618

Table S10. Intermolecular Interaction Energies with Electron Density from crystal Explorer.

System	N	Symop	R (Å)	Electron Density	E_{ele}	E_{pol}	E_{dis}	E_{rep}	E_{tot}
CAF·4HBA·H_2O	0	-	6.61	B3LYP/6-31G(d,p)	-78.8	-17.1	-15.3	94.1	-51.2
CAF	1	-x, -y, -z	7.68	B3LYP/6-31G(d,p)	-9.7	-1.6	-4.3	0.5	-14.5
CAF	1	x, -y+1/2, z+1/2	7.63	B3LYP/6-31G(d,p)	-11.4	-2.5	-6.8	2.0	-17.7
CAF	1	x, y, z	3.97	B3LYP/6-31G(d,p)	28.1	-10.9	-41.7	14.0	-4.6
CAF	0	x, -y+1/2, z+1/2	8.94	B3LYP/6-31G(d,p)	3.7	-2.8	-4.3	4.3	1.5
CAF	1	-x, y+1/2, -z+1/2	9.08	B3LYP/6-31G(d,p)	3.5	-0.4	-2.2	0.8	2.0

CAF	1	-x, -y, -z	8.49	B3LYP/6-31G(d,p)	-1.4	-2.2	-4.8	2.8	-4.9
CAF	0	-	6.31	B3LYP/6-31G(d,p)	-0.2	-0.0	-0.1	0.0	-0.3
CAF	0	-	4.96	B3LYP/6-31G(d,p)	3.5	-0.4	-2.2	0.8	2.0

Table S11: Intermolecular Interaction Energies with Electron Density from Cp2k.

	Total Energy
sp	-464.4
opt	-464.35
gas phase 1 (4HBA)	-91.49
gas phase 2 (water)	-17.22
gas phase 3 (caffeine)	-123.35
E_{tot}	-77.26
